# Elucidating Polyphosphate Anion Binding to Lanthanide Complexes Using EXAFS and Pulsed EPR Spectroscopy

**DOI:** 10.1021/acs.inorgchem.4c03399

**Published:** 2024-10-16

**Authors:** Hannah K. Pyle, Martyna Judd, Anthony Barancewicz, Alexander J. Mayer, Nicholas Cox, Simon A. Kondrat, Stephen J. Butler

**Affiliations:** † Department of Chemistry, Loughborough University Epinal Way, Loughborough LE11 3TU, United Kingdom; ‡ Research School of Chemistry The Australian National University Canberra, ACT 2605, Australia

## Abstract

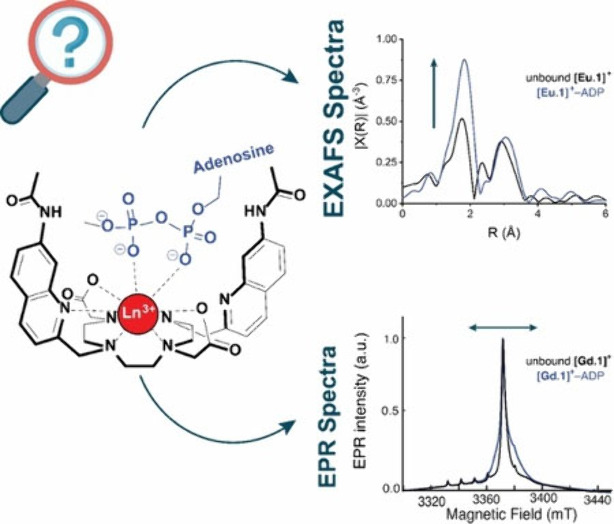

Reversible anion binding to lanthanide complexes in aqueous solution has emerged as an effective method for anion sensing. Through careful design of the organic ligand, luminescent lanthanide complexes capable of binding biologically relevant anions in a bidentate or monodentate manner can be realized. While single-crystal X-ray diffraction analyses and NMR spectroscopy have revealed the structural geometry of several host–guest complexes, the challenge remains in designing preorganized lanthanide receptors with enhanced anion selectivity for broader applications in diagnostics and bioimaging. To address this challenge, innovative and complementary methods to investigate host-anion binding geometry are becoming increasingly important. Herein, we demonstrate the combined use of Eu L_3_-edge extended X-ray absorption fine structure (EXAFS) and electron paramagnetic resonance (EPR) spectroscopy to elucidate the binding of nucleoside phosphates (ATP, ADP, and AMP) to a cationic lanthanide complex. We establish that ATP unequivocally binds the lanthanide center in a bidentate manner in water, while ADP adopts both bidentate and monodentate modes, and AMP binds in a monodentate manner. This interdisciplinary approach provides deeper insight into lanthanide host–guest chemistry in solution, laying the groundwork for designing emissive probes that undergo specific anion-induced structural changes and elicit desired optical responses upon binding.

## Abstract 2

We present a novel approach combining extended X-ray absorption fine structure (EXAFS) and electron paramagnetic resonance (EPR) spectroscopy to analyze the binding of nucleoside phosphate anions (ATP, ADP, and AMP) to lanthanide complexes in water. Our study uncovers distinct anion binding modes, offering valuable insights for designing selective lanthanide-based receptors with applications in sensing and bioimaging.

## Introduction

Supramolecular receptors that reversibly bind and sense biological anions show great potential as innovative tools for bioassays, diagnostics and cellular imaging to facilitate biomedical and clinical research.^1−5^ The design and synthesis of molecular receptors that operate in water and exhibit high anion selectivity remains a significant challenge.^1,6,7^ The selective recognition of larger biological anions generally requires the synthesis of larger and more intricate host architectures bearing multiple converging recognition motifs to engage their target. To optimize the receptor design, complementary spectroscopic methods are needed to characterize the structural configuration and geometry of the host–guest species under the conditions in which the receptor is to be used, e.g., in buffered aqueous solution.

Luminescent lanthanide complexes have emerged as an effective class of anion receptors.^8−10^ They offer advantages for biological sensing and imaging due to their unique optical properties, including (1) long emission lifetimes, which enable time-gated measurements to eliminate background autofluorescence, and (2) sharp line-like emission bands that can be monitored ratiometrically.^11−13^ Several lanthanide complexes have been developed capable of selective anion recognition, achieved by carefully considering factors such as organic ligand structure and its conformational flexibility, the steric hindrance at the metal center, and the overall charge of the complex.^9,14−18^

The binding configuration of certain anions to lanthanide complexes has been determined by single-crystal X-ray diffraction (XRD) and/or NMR spectroscopic methods, with both bidentate and monodentate binding modes identified. However, reliance upon these characterization methods has limitations: a single-crystal XRD structure does not necessarily reflect the host–guest structure in the solution, whereas NMR spectra of dynamic host–guest interactions involving paramagnetic centers often show significant exchange broadened signals that complicate their interpretation.

**Figure 1:**
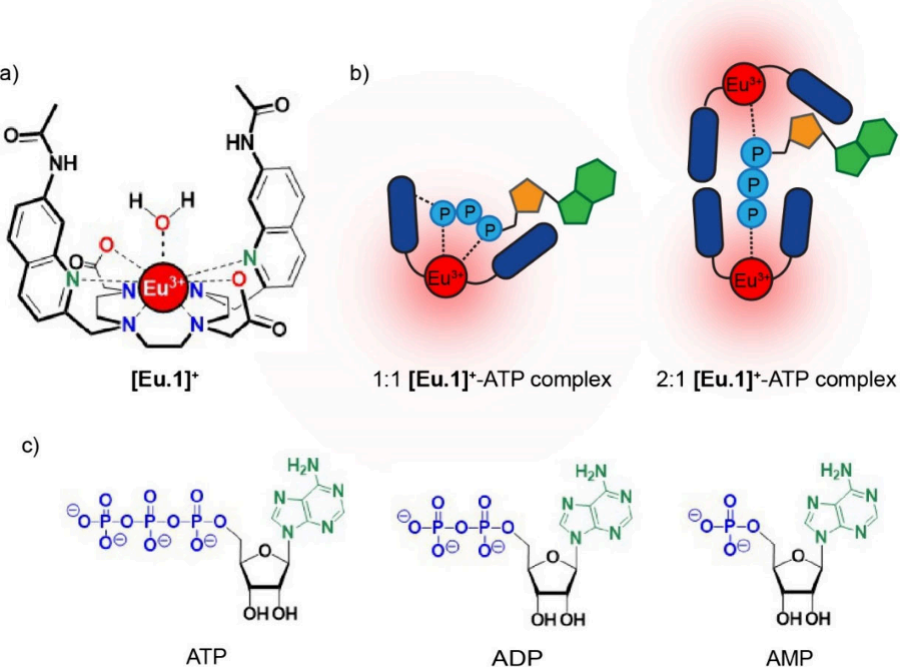
(a) Structure of monoaqua complex **[Eu.1]^+^** showing oxygen and nitrogen donor atoms in color. (b) Illustrations depicting two possible binding geometries of ATP to **[Eu.1]^+^**. (c) Structures of biological anions ATP, ADP, and AMP.

We previously showed that the macrocyclic europium complex **[Eu.1]**^**+**^ (Figure 1a) has sufficient flexibility to accommodate the nucleoside phosphate anions ADP or ATP within the cavity defined by the two quinoline amide arms.^19,20^ Upon binding each anion, the coordinated water molecule is released, causing a large enhancement in Eu(III) emission intensity and lifetime. Similar binding affinities and changes in emission shape were observed for ATP and ADP, especially within the Δ*J* = 1 emission band (582–605 nm), indicating similar coordination environments at the europium ion (Figure S2). However, because only the most emissive species is observed in the spectrum, the existence of more than one host–guest species could not be ruled out. ^31^P NMR spectroscopy of the **[Eu.1]**^**+**^–ATP adduct suggested a bidentate binding mode for ATP via the α- and γ-phosphate groups (Figure 1b). However, the NMR data were also consistent with a 2:1 sandwich-like host–guest structure that engages the α- and γ-phosphates (Figure 1b).^21^ Furthermore, the ADP binding mode could not be determined due to the exchange broadened signals in the NMR spectrum. **[Eu.1]**^**+**^ showed weak binding to AMP, resulting in a much smaller emission increase and a distinctive spectral shape compared with that observed for ATP and ADP, indicating a monodentate binding mode (Figure S1).

Considering the limitations of NMR and emission spectroscopy described above, we sought new methods to characterize host–guest interactions of lanthanide complexes: extended X-ray absorption fine structure (EXAFS) and electron paramagnetic resonance (EPR). Importantly, these two complementary techniques allow the characterization of the host–guest complex in solution. Because both techniques directly measure the host lanthanide ion, as opposed to the guest (as in NMR), they are particularly sensitive to the coordination number and environment of the metal ion itself. Eu L_3_-edge EXAFS, a form of X-ray absorption spectroscopy, probes the local coordination environment of the europium ion through the interpretation of emitted photoelectron interference patterns. Meanwhile, EPR spectroscopy offers insight into paramagnetic species’ electronic and magnetic properties. For EPR analysis, we used the homologous gadolinium complex **[Gd.1]**^**+**^. Gadolinium(III) is used due to its favorable magnetic properties: it is both high spin (*S* = ^7^/_2_) and orbitally nondegenerate, with a half-filled valence f-shell (4f^7^) electronic configuration. At high magnetic fields (W-band, 3.4 T), this leads to Gd(III) complexes displaying an intense, narrow central EPR line, ideal for sensitive detection of its local environment via double resonance techniques such as electron nuclear double resonance (ENDOR).

Herein, we present the advantages of combining EPR and EXAFS techniques to elucidate lanthanide-based host–guest interactions. A combined EPR and EXAFS approach has been used previously to provide mechanistic insights into metal catalysts,^22^ but to the best of our knowledge, it has not been commonly applied to examining supramolecular host–guest binding. We show that EXAFS unambiguously provides coordination numbers, especially for the dominant host–guest species at ambient temperatures, yielding element-specific ligand counts. Meanwhile, EPR/ENDOR can definitively identify ligand substrates, even under substoichiometric binding conditions, and can potentially track and quantify anion binding. Here, we note that the probe concentrations used in the EPR experiments (low micromolar) are comparable to those used in cellular imaging. We further show that EPR line-shape analysis and EXAFS address host–guest-induced conformational changes, providing structural constraints for density functional theory (DFT) models. By leveraging the complementary nature of these techniques, we have gained a deeper understanding of lanthanide-based host–guest interactions, thereby facilitating the design of next-generation lanthanide probes with improved selectivity for polyphosphate anions.

## Results and Discussion

### EXAFS Analysis of Monoaqua Europium Complex **[Eu.1]^+^**

EXAFS offers atomic-level structural information, enabling distances and coordination numbers of atoms around the Eu(III) center to be determined. Importantly, EXAFS can be applied to noncrystalline or solution samples to provide structural information in realistic environments.^23^ It has been used, in combination with *ab initio* molecular dynamics or DFT simulations, to study ligand and solvent molecule coordination, the stability of discrete europium complexes, and the incorporation and preservation of europium in different materials.^24,25^

Initially, we used EXAFS to study a solid sample of the monoaqua europium complex **[Eu.1]**^**+**^, which allowed a model to be developed for the coordination environment of the Eu complex in the absence of anions. These models are informed by previously determined single-crystal XRD data and DFT simulation of the **[Eu.1]**^**+**^ complex. The solid powder provided the best quality spectroscopic data due to the relatively high Eu concentration and reduced structural disorder relative to the complex in solution caused by both structural flexibility and complex secondary solvation shells.^24,25^

**Figure 2:**
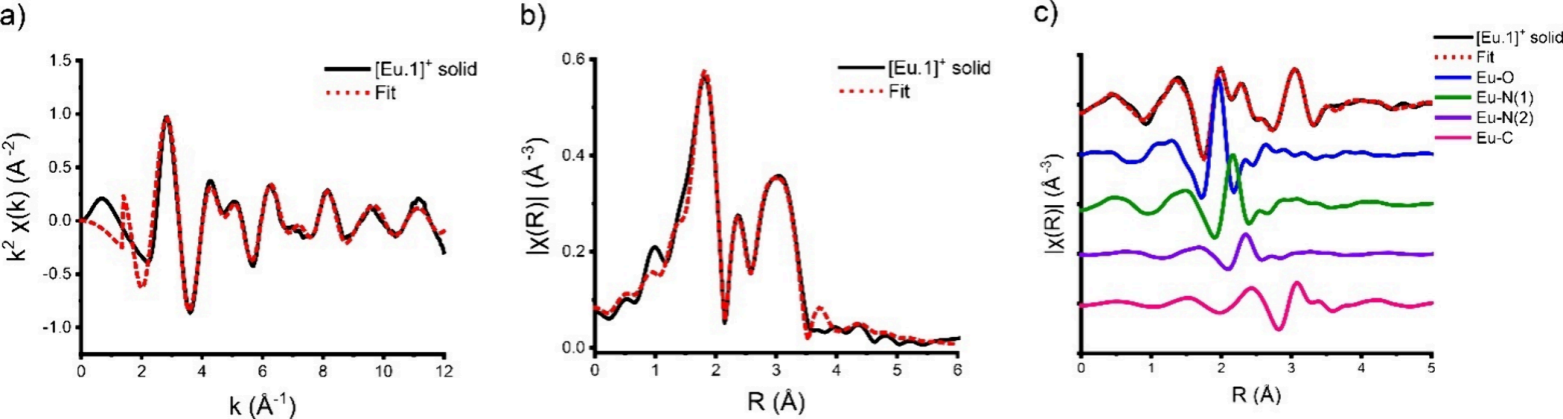
(a) Measured *k*^2^χ(*k*) (Å^–2^), (b) magnitude of the Fourier transform of *k*^2^χ(*k*) (Å^–2^), and (c) real χ(*R*) data with fitted paths of the **[Eu.1]**^**+**^ solid sample. Only the predominant Eu–C paths are shown for clarity.

Figure 2a shows the measured *k*-space data with the fit, illustrating the EXAFS oscillations essential for determining the structural parameters. The nonphase-corrected magnitude of the Fourier transform data (Figure 2b) provides information analogous to a pseudoradial distribution plot of species around the Eu(III) center, while Figure 2c shows the *R*-space data with the fit, along with the individual relevant paths. These include the Eu–O, Eu–N(1), and Eu–N(2) paths and one predominant Eu–C path for clarity. *q*-space data and associated fits are shown in the Supporting Information (SI). As verified by EXAFS data fitting (*vida infra*), the primary peak observed at approximately 1.9 Å corresponds to the near-neighbor Eu–O coordination, while the features appearing between 2.0 to 2.4 Å are associated with paths from near-neighbor Eu–N coordination. The Eu–O and Eu–N paths are out of phase, while features higher in *R* relate to multiple Eu–C scattering paths.

As described in the Experimental Section, data fitting was performed using fixed coordination numbers determined by the XRD data.^21^ Model 1, with a coordination number of 3 for Eu–O (i.e., two carboxylates and one water molecule) and two paths Eu–N (i.e., macrocyclic and quinoline nitrogen atoms), was used to fit the data. Table 1 reveals that all relevant fitted parameters were realistic, with acceptable 2σ^2^ values (i.e., >0 and <0.02 Å^2^), *R*_factors_ (<0.05), χ^2^ fitting values (<400), and calculated path lengths that closely aligned with the X-ray crystal structure of the formate adduct of **[Eu.1]**^**+**^ (Figure S3).^21^ Some minor differences relative to the diffraction data were observed. Specifically, the average Eu–O bond length was extended by 0.04 Å, while the Eu–N(1) and Eu–N(2) lengths were shortened by 0.04 Å. Notably, similar trends of shorter distances observed by diffraction relative to EXAFS have been reported for a range of other lanthanide and actinide complexes.^25,26^ To further verify the model, the Eu–O coordination number was floated and found to be within error of the expected value of 3. These small variations indicate very good agreement between the fitted model and the crystallographic data.^21^

**Table 1:** EXAFS Plotting Parameters for **[Eu.1]**^**+**^ (Solid, Solution at 77 K, and Solution at 293 K)

sample	path	*N*^a^	*R* (Å)^b^	2σ^2^ (Å^2^)^b^	*E*_f_ (eV)^b^	*R*_factor_ [χ^2^]^c^
solid **[Eu.1]^+^**	Eu–O	3	2.38(1)	0.0021(1)	1.4(4)	0.014 [326]
	Eu–N(1)	4	2.61(1)	0.006(4)		
	Eu–N(2)	2	2.78(4)	0.007^d^		
solid **[Eu.1]^+^** floating Eu–O CN^e^	Eu–O (CN float)	3.1(5)	2.38(1)	0.0021^e^	1.3(4)	0.015 [340]
**[Eu.1]^+^** at77 K	Eu–O	3	2.39(1)	0.0022(1)	7(1)	0.019 [72]
	Eu–N(1)	4	2.61(2)	0.006(7)		
	Eu–N(2)	2	2.77(7)	0.0064^d^		
**[Eu.1]^+^** at 293 K	Eu–O	3	2.40(2)	0.004(1)	7(1)	0.040 [78]
	Eu–N(1)	4	2.63(2)	0.006^d^		
	Eu–N(2)	2	2.82(5)	0.007^d^		

a *N* = coordination number.

b Uncertainty in the interatomic distances, Debye–Waller factors, and *E*_0_ values is given in parentheses for the last decimal place.

c Calculated χ^2^ values in parentheses.

d Path defined at Eu–N1 × 1.1 during the fit.

e Comparison fit with Eu–O CN floated and 2σ^2^ fixed; no changes in the Eu–N paths were observed, and these values have been omitted for clarity. *S*_0_^2^ is set at 1. *K* fitting range of 3–11 Å^–1^ and *R* fitting range of 1–4 Å. Full fitting data, including Eu–C paths, are shown in Table S2.

As noted by Smerigan et al., Eu–C paths require fitting to improve the statistical quality of the fit,^24^ yet due to the required constraints during fitting, provide little additional information and are therefore not shown for simplicity (fitting data including these paths are shown in Table S2).

Next, we measured EXAFS of a 10 mM solution of **[Eu.1]**^**+**^ in HEPES buffer (10 mM, pH 7.0). The sample was frozen at 77 K to reduce the structural disorder due to the greater structural flexibility of the complex in solution. Using model 1, the EXAFS data from the solution at 77 K were successfully fitted (Figure S6). A high 2σ^2^ error associated with Eu–N(1) reflects the increased challenge in achieving high-quality fitting compared with the solid sample. Notably, the fitting parameters for the Eu–O path remained comparable to that of the recorded solid, which was considered realistic regarding the 2σ^2^ value and bond length (*vide supra*).

Fixing the Eu–N(1) 2σ^2^ value to 0.006 Å^2^ resulted in a stable fit with no significant impact on the Eu–O parameters [2σ^2^= 0.0022(7) Å^2^ and *R* = 2.40(2) Å]. Finally, EXAFS of the **[Eu.1]**^**+**^ solution at room temperature could be satisfactorily fitted to model 1 (fit shown in Figure S7), with the Eu–N(1) path 2σ^2^ value fixed at 0.006 Å^2^. Subtle changes in the Eu–O path fitting are observed, leading to a 2σ^2^ of 0.004(1) Å^2^, indicating slightly more disorder due to the increased measurement temperature.

In summary, the EXAFS data indicate that the local structure of **[Eu.1]**^**+**^ remains essentially unchanged, whether in the solid or solution phase and at both room temperature and 77 K. Additionally, our model effectively fits the complex across all states and temperatures. While the model we used remains valid and robust for interrogation of Eu–O path changes, caution should be taken when considering the influence of added ATP or ADP when assessing changes in Eu–N coordination.

### EXAFS Analysis of **[Eu.1]^+^** Binding to ATP or ADP at 77 K

**Figure 3:**
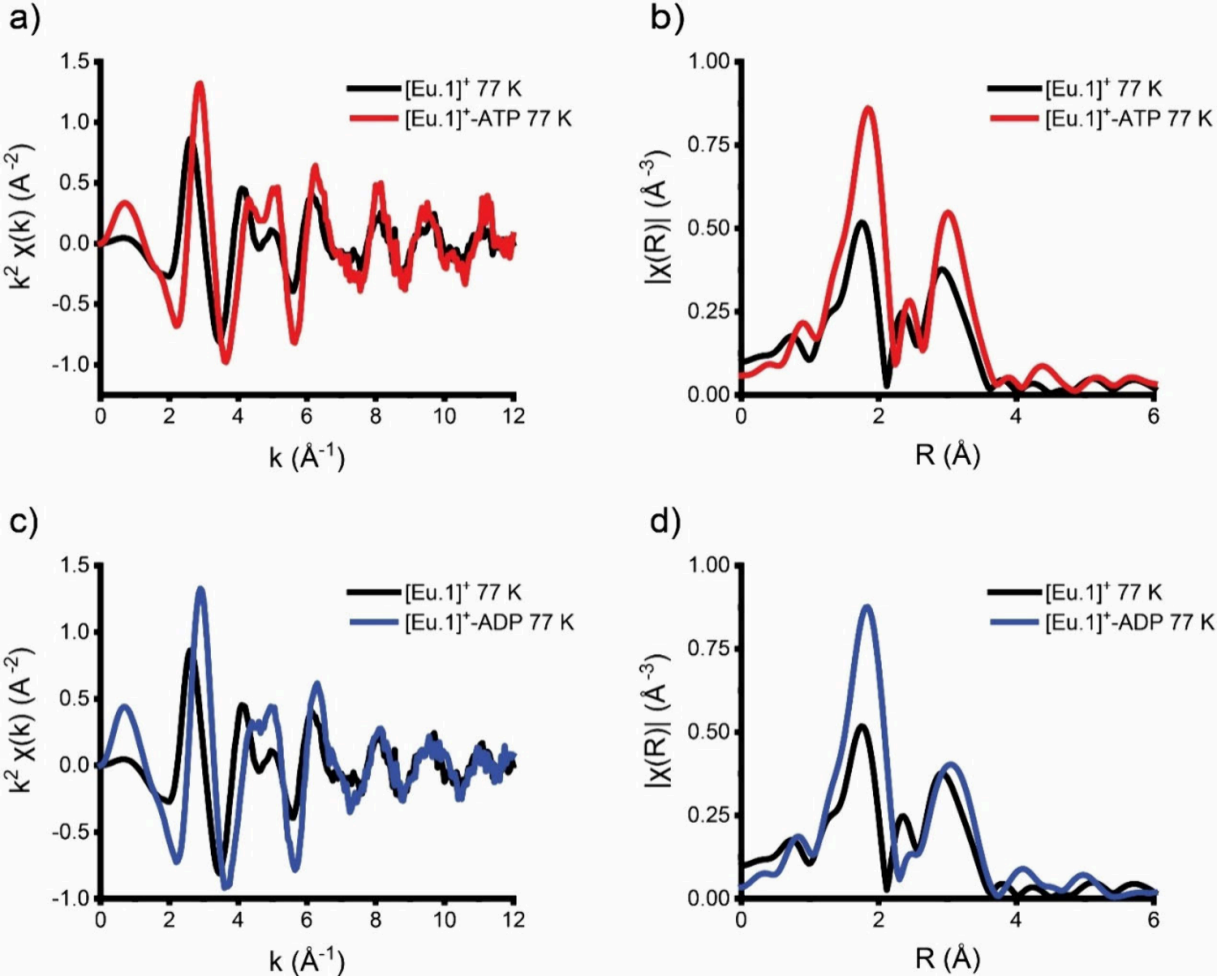
(a) Comparison of *k*^2^χ(*k*) (Å^–2^), (b) Fourier transforms of *k*^2^χ(*k*) (Å^–2^) of **[Eu.1]**^**+**^ solution at 77 K (black) and with added ATP (red), (c) comparison of *k*^2^χ(*k*) (Å^–2^), and (d) Fourier transforms of *k*^2^χ(*k*) (Å^–2^) of **[Eu.1]**^**+**^ solution at 77 K (black) and with added ADP (blue).

Initial comparison of the *k*- and *R*-space data of **[Eu.1]**^**+**^ with and without ATP in HEPES-buffered solution (10 mM, pH 7.0) at 77 K revealed significant changes in the local Eu(III) environment (Figure 3). Notable changes in the phasing of the *k*-space data (Figure 3a) suggest changes in path lengths while increased intensities of Eu–O and Eu–C/second shell paths in the magnitude of the Fourier transform were observed in the presence of ATP. Changes in the fine structure associated with features above 3 Å are challenging to accurately model, as noted previously, but can be speculated to be associated with disruption of the solvation sphere by nucleoside phosphate anion. To further interpret these changes, innovative modeling approaches, such as coupled EXAFS and molecular dynamic simulations used by Summers et al., could be employed.^24^ Fitting the first shell EXAFS data of **[Eu.1]**^**+**^–ATP using the monoaqua **[Eu.1]**^**+**^ model 1 was unsatisfactory (Table 2), as was expected from the pronounced changes seen in the *k*-space data (particularly at low *k*). The errors in the Eu–N path fitting were exceptionally large with an error greater than the value (0.02 ± 0.1) and defied physical plausibility.

**Table 2:** EXAFS Plotting Parameters for **[Eu.1]**^**+**^–ATP EXAFS Data at 77 K and **[Eu.1]**^**+**^–ADP EXAFS Data at 77 K^a^

entry	path	*N*^b^	*R* (Å)^c^	2σ^2^ (Å^2^)^c^	*E*_f_ (eV)^c^	*R*_factor_ [χ^2^]^d^
**[Eu.1]^+^**–ATP	Eu–O	3	2.39(1)	0.001(1)	8(3)	0.059 [255]
model 1	Eu–N(1)	4	2.58(78)	0.02(10)		
	Eu–N(2)	2	2(2)	0.021^e^		
**[Eu.1]^+^**–ATP	Eu–O	4	2.38(6)	0.0024(1)	7(1)	0.026 [148]
model 2	Eu–N(1)	4	2.62(4)	0.013(5)		
**[Eu.1]^+^**–ADP	Eu–O	3	2.31(1)	0.0006(27)	5(1)	0.027 [159]
model 1	Eu–N(1)	4	2.47(3)	–0.001(3)		
	Eu–N(2)	2	2.65(3)	n/a		
**[Eu.1]^+^**–ADP	Eu–O	4	2.36(2)	0.0038(4)	5(2)	0.034 [199]
model 2	Eu–N(1)	4	2.66(6)	0.007(16)		

a Model 1 utilizes **[Eu.1]**^**+**^ solid fitting parameters, while model 2 represents a bidentate anion binding model.

b *N* = coordination number.

c Uncertainty in the interatomic distances, Debye–Waller factors, and *E*_0_ values is given in parentheses for the last decimal place.

d Calculated χ^2^ values in parentheses.

e Path defined at Eu–N1 × 1.1 during the fit. *S*_0_^2^ is set at 1. *K* fitting range of 3–11 Å^–1^ and *R* fitting range of 1–4 Å. Full fitting data, including Eu–C paths, are shown in Table S3.

Furthermore, the 2σ^2^ value associated with the Eu–O path was essentially reduced to zero, a physically untenable condition. This unrealistic disorder parameter possibly stems from the fit’s attempt to compensate for the undercoordination of the Eu–O paths, i.e., the actual coordination of Eu–O is greater than 3 when ATP is bound. This strongly suggests that ATP does not adopt a monodentate binding mode at the Eu(III) center, such as the 2:1 sandwich-type structure shown in Figure 1b.

To confirm the bidentate binding of ATP (Figure 1b) and rule out a 2:1 host–guest binding mode, an alternative model (model 2) was tested (Table S3), which increases the Eu–O coordination number from 3 to 4. Comparisons of best fits in *k*-, *R*- and *q*-space with experimental data are shown in Figure S8. Model 2 provided a significantly improved fit, giving viable Eu–O path parameters with realistic 2σ^2^ values (>0 and <0.02 Å^2^) and a more reasonable fit for Eu–N(1), as shown in Table 2. Efforts were made to reduce the Eu–N(2) coordination number from 2 to 1, but without satisfactory fitting (Table S3). Complete exclusion of the Eu–N(2) path was required to prevent convergence with Eu–N(1) (Table 2). This observation suggests that the disorder in the Eu–N(2) path, possibly caused by the motion of the quinoline arms, is so pronounced that it becomes essentially unresolved through EXAFS.

Despite the observable changes in the *k*-space (at low *k*) and *R*-space data, no discernible differences in Eu–C 2σ^2^ (beyond the error) could be seen between **[Eu.1]**^**+**^ [0.007(1) Å^2^] and **[Eu.1]**^**+**^–ATP [0.004(2) Å^2^]. As descibed above, a combination of a simplified Eu–C fitting model and the relatively small predicted change in the average Eu–C distances has resulted in the extent of changes not being captured by the fitting model. It is clear from the phasing in low *k*-space data that new light scattering paths are present, which could include **[Eu.1]**^**+**^–solvent or first shell **[Eu.1]**^**+**^–P paths. While compelling to fit and ascertain a coordination number for Eu–P paths and thus strengthen the bidentate binding mode model (Eu–P CN = 2, supporting bidentate binding), these paths could not be accurately modeled. Specifically, as shown in Table S5, Eu–P paths could be applied to **[Eu.1]**^**+**^ without ATP or ADP present, and with no significant change in the quality of fit, thus rendering application of this path to binding models 1 or 2 impracticable.

Taken together, Eu L_3_-edge EXAFS demonstrates bidentate binding of ATP to the Eu(III) ion, coupled with a discernible alteration in the quinoline nitrogen environment surrounding the europium ion. The EXAFS data are consistent with previous DFT optimizations of the **[Eu.1]**^**+**^–ATP complex, which indicated the dissociation of one quinoline arm to accommodate the sterically bulky anion.^21^

Next, we applied our models to shed light on the more ambiguous binding of ADP. Previous ^31^P NMR spectral data of the **[Eu.1]**^**+**^–ADP complex showed exchange-broadened signals, so the binding mode could not be determined.^21^ Fitting the EXAFS data of **[Eu.1]**^**+**^–ADP at 77 K using model 1 was similarly unsuccessful to that seen with **[Eu.1]**^**+**^–ATP, whereas a suitable fit for ADP binding was achieved using bidentate model 2. This is significant because it provides the first primary evidence that ADP binds in a bidentate manner. When comparing the bidentate model EXAFS fitting of **[Eu.1]**^**+**^–ADP with **[Eu.1]**^**+**^–ATP, we observed a higher Eu–O 2σ^2^ value for the former adduct [0.0038(4) Å^2^ versus 0.0024(1) Å^2^], which indicates subtle differences in the local structure at the Eu(III) center. Given that 2σ^2^ and coordination number fitting may be correlated in EXAFS fitting when there is insufficiently long *k*-space data and interference from multiple paths, as is the case here, it is difficult to distinguish between these parameters’ changes. Therefore, the result indicates bidentate binding of ADP, but with increased structural disorder compared with ATP. This could be due to the asymmetric binding of ADP or the greater motion of the host–guest species. Alternatively, it could indicate that ADP binds in two distinct conformations, predominantly bidentate with a minority monodentate. However, because both values of 2σ^2^ are reasonable, the EXAFS does not support the scenario of a mixture of binding modes with substantial proportions of the monodentate complex. Overall, it is clear that the **[Eu.1]**^**+**^–ADP complex primarily involves bidentate binding via α- and β-phosphates, albeit with greater disorder compared with ATP.

### EXAFS Analysis of **[Eu.1]^+^** Binding to ATP or ADP at 293 K

**Figure 4:**
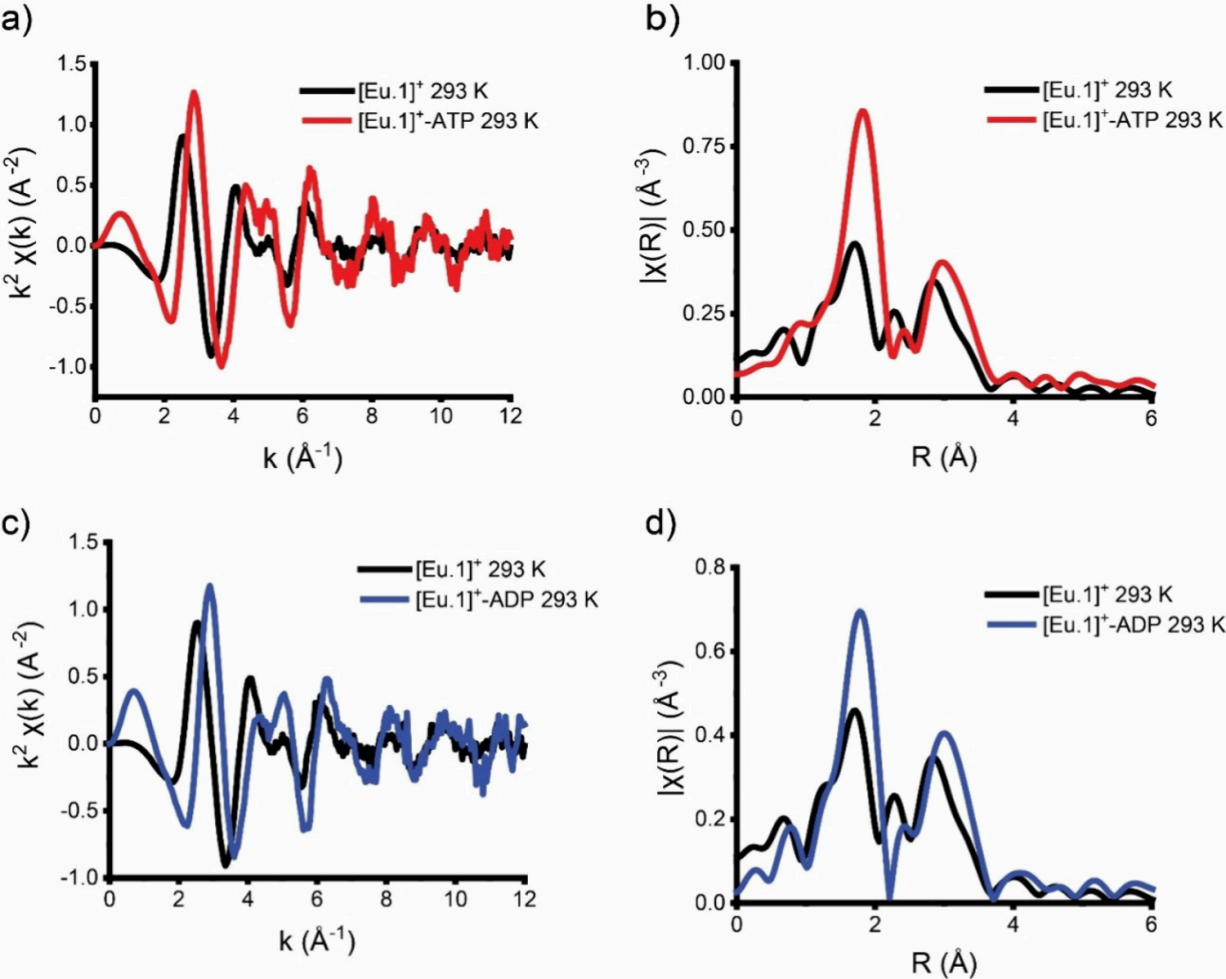
(a) Comparison of *k*^2^χ(*k*) (Å^–1^), (b) Fourier transforms of *k*^2^χ(k) (Å^–1^) of **[Eu.1]**^**+**^ alone (black) and in the presence of ATP (red), (c) comparison of *k*^2^χ(*k*) (Å^–1^), and (d) Fourier transforms of *k*^2^χ(*k*) (Å^–1^) of **[Eu.1]**^**+**^ alone (black) and in the presence of ADP (blue).

Finally, the solution of **[Eu.1]**^**+**^–ADP was examined at room temperature and compared with that obtained for **[Eu.1]**^**+**^–ATP under the same conditions. Notable differences in the magnitude of the Fourier transform data were observed (Figure 4). Specifically, the intensity of the first feature corresponding to Eu–O coordination reached its highest value for the ATP-bound complex, an intermediate level for the ADP-bound complex, and the lowest intensity for the monoaqua Eu complex. EXAFS fitting of the **[Eu.1]**^**+**^–ATP sample at 293 K (Tables 3 and S4 and Figures S8 and S9) gave comparable results to the frozen sample at 77 K using model 2, which simulates bidentate ATP binding and provides a more realistic fit than model 1 (monodentate binding). The EXAFS data for **[Eu.1]**^**+**^–ADP at 293 K also fitted satisfactorily, assuming bidentate coordination. We observed a significant difference in 2σ^2^ values, with 0.006(1) Å^2^ for ATP binding and 0.003(1) Å^2^ for ADP binding, which again reflects differences between the disorder of each. This is unsurprising, as molecular motion will be more significant at 293 K. The notably larger 2σ^2^ accounts for the visible differences in the magnitude of the Fourier transform data seen in Figure 4. Therefore, the EXAFS data at both 77 and 293 K confirm the bidentate binding of both ATP and ADP, but significantly more structural disorder was observed for the latter.

**Table 3:** EXAFS Plotting Parameters for **[Eu.1]**^**+**^–ATP and **[Eu.1]**^**+**^–ADP EXAFS Data at 293 K^a^

entry	path	*N*^b^	*R* (Å)^c^	Δσ^2^ (Å^2^)^c^	*E*_f_ (eV)^c^	*R*_factor_ [χ^2^]^d^
**[Eu.1]^+^**–ATP	Eu–O	3	2.34(2)	0.0002(32)	7(2)	0.036 [81]
model 1	Eu–N(1)	4	2.51(3)	0.00024(52)		
	Eu–N(2)	2	2.70(5)	0.00027^e^		
**[Eu.1]^+^**–ATP	Eu–O	4	2.38(2)	0.003(1)	7(2)	0.039 [88]
model 2	Eu–N(1)	4	2.59(3)	0.008(4)		
**[Eu.1]^+^**–ADP	Eu–O	3	2.33	0.0002(14)	6(1)	0.036 [57]
model 1	Eu–N(1)	4	2.52	0.0002(20)		
	Eu–N(2)	2	2.73	0.00025^b^		
**[Eu.1]^+^**–ADP	Eu–O	4	2.36(2)	0.004(1)	5(2)	0.050 [75]
model 2	Eu–N(1)	4	2.59(4)	0.010(5)		

a Model 1 utilizes **[Eu.1]**^**+**^ solid fitting parameters, while model 2 represents a bidentate anion binding model.

b *N* = coordination number.

c Uncertainty in the interatomic distances, Debye–Waller factors, and *E*_0_ values is given in parentheses for the last decimal place.

d Calculated χ^2^ values in parentheses.

e Path defined at Eu–N1 × 1.1 during the fit. *S*_0_^2^ is set at 1. *K* fitting range of 3–11 Å^–1^ and *R* fitting range of 1–4 Å. Full fitting data, including Eu–C paths, are shown in Table S4.

### EPR Analysis–EPR Line Shape Data

**Figure 5:**
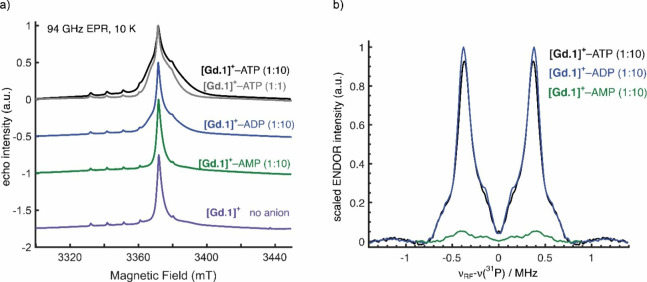
(a) Echo-detected field-sweep spectra of the Gd(III) EPR line for **[Gd.1]**^**+**^ without anion binding, compared to binding with ATP, ADP, or AMP in 1:10 Gd(III) to anion concentrations. (b) Signal-to-noise comparison of the ^31^P ENDOR spectra of ATP-, ADP-, and AMP-bound **[Gd.1]**^**+**^. Spectra are scaled to the echo intensity, i.e., the ENDOR response as a fraction of the EPR signal.

To further investigate the structure and disorder of the **[Eu.1]**^**+**^–ADP/ATP adducts in solution, we turned to EPR spectroscopy. The homologous gadolinium complex **[Gd.1]**^**+**^ was used for EPR analysis, as described in the Introduction. Qualitatively, the 94 GHz (W-band) EPR line-shape data (Figure 5a) support the difference in binding modes and affinities of **[Gd.1]**^**+**^ with ATP, ADP, and AMP.

Complex **[Gd.1]**^**+**^ without the addition of a guest ligand exhibits a narrow central ⟨−^1^/_2_ ↔ ^1^/_2_⟩ transition. This indicates that the complex’s zero-field splitting (ZFS, **D**-tensor) is small, estimated to be around ±50 MHz (∼0.002 cm^–1^; Figure S13). A small ZFS is a marker for a spherically symmetric ligand field. Adding AMP did not significantly alter the EPR line shape, suggesting that the binding affinity of AMP is low, as seen for the analogous complex **[Eu.1]**^**+**^.^19^

The addition of ADP and ATP did, however, change the EPR spectrum, specifically broadening of the central ⟨−^1^/_2_ ↔ ^1^/_2_⟩ transition (Figure 5a). This is due to an increase in the ZFS of the complex, as all other mechanisms could be excluded (see Figures S14 and S15 showing ENDOR measurements performed on the high-field edge of the EPR spectrum to exclude contributions from transitions between lower *m*_s_ levels). In their quantitative study of ZFS parameter distributions in Gd(III) complexes, Clayton et al. have shown that larger ZFS values and distributions generally correlate with greater asymmetry of the Gd(III) coordination environment,^27^ and as such, it can be inferred that the overall symmetry of the complex must be lowered upon ADP/ATP binding, consistent with **[Eu.1]**^**+**^ EXAFS data. Unfortunately, we could not achieve robust simulations of the broadened spectra of **[Gd.1]**^**+**^–ATP and **[Gd.1]**^**+**^–ADP to quantify changes in the ZFS parameters. However, we note that the ZFS parameters for both complexes must have a large distribution (strain) consistent with a significant degree of disorder, i.e., variation in the pendant quinoline arms, again in line with the EXAFS results. To address the nature of the ligand binding mode of ADP and ATP, we turned to ^31^P Mims ENDOR.

### ^31^P Mims ENDOR Intensities and Line Shape

Figure 5 shows the ^31^P Mims ENDOR spectra measured at 94 GHz (W-band). These represent the sum of multiple measurements, which differ in terms of the first interpulse delay (τ-dependence) to suppress blind-spotting artifacts.^28^ The ENDOR intensities shown indicate the proportion of ^31^P species bound to the **[Gd.1]**^**+**^ complex. Note that the ENDOR experiment only detects the Gd(III)-bound substrate. Unbound nucleoside phosphate, where the ^31^P is distant from the Gd(III), does not contribute any significant ENDOR intensity, since the ENDOR intensity scales with the distance, *r*, inversely as 1/*r*^6^.^29^ The baseline of the ENDOR spectrum is simply the EPR echo intensity without the effect of a resonant RF pulse. Therefore, the relative binding affinities of the different nucleoside phosphates can be determined by scaling the ENDOR intensities to the baseline echo intensity.

**Figure 6:**
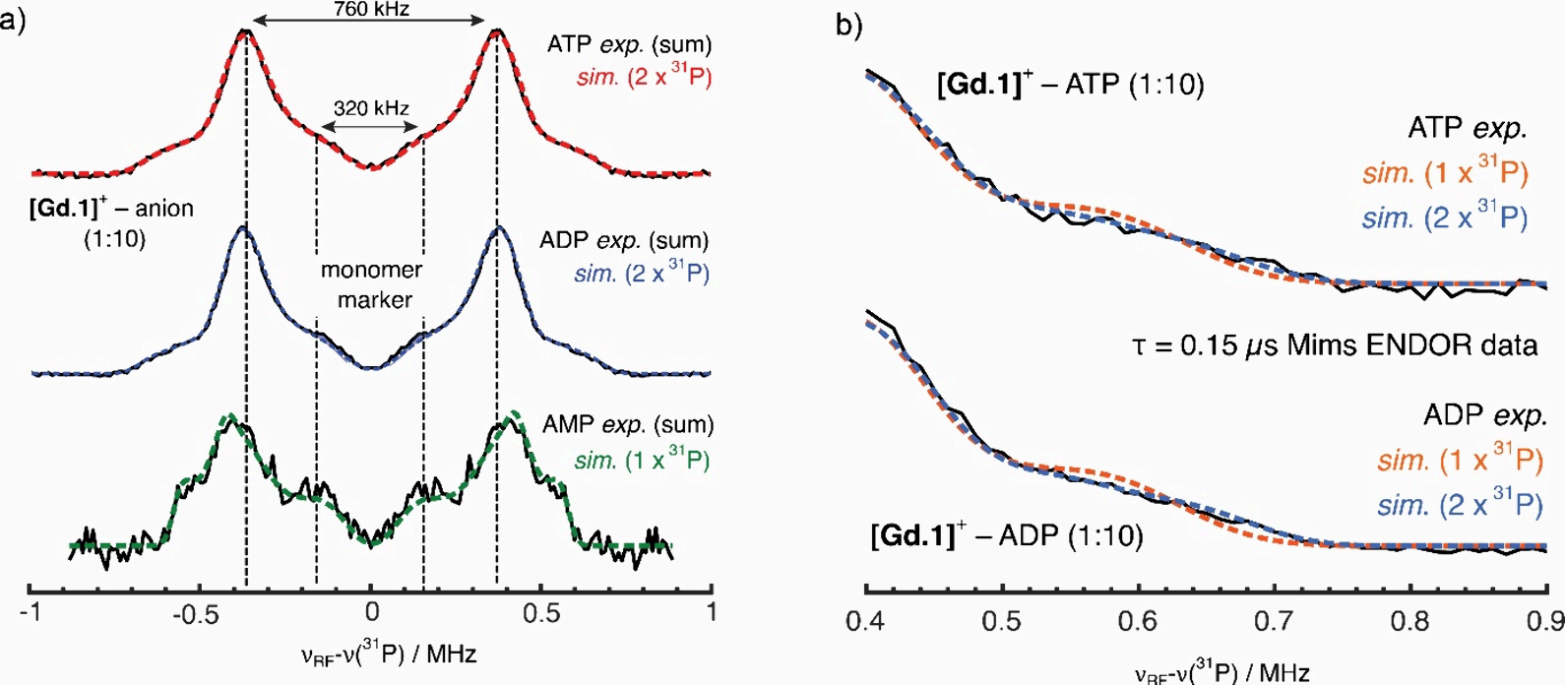
Experimental ^31^P ENDOR data and fits of **[Gd.1]**^**+**^ anion adducts. (a) ENDOR data (black traces) and simulations (colored traces) of **[Gd.1]**^**+**^ in the presence of ATP, ADP, and AMP in a 1:10 molar ratio. The spectra of each complex were measured at different interpulse delay times τ, with the sums of these spectra displayed in the figure. The fitting procedure minimizes the fit to all τ-dependent data simultaneously. The displayed fits are the best-fit bidentate (ATP and ADP) or monodentate (AMP) models. (b) Outer shoulder region of the experimental **[Gd.1]**^**+**^–ATP and **[Gd.1]**^**+**^–ADP data, comparing the performance of the 1 × ^31^P and 2 × ^31^P fits. In panel a, the ±160 kHz central shoulder is marked. Compared to the main peak at ±380 kHz, its relative intensity can be considered a marker for monodentate binding (blue-shaded region). The increase in this feature in the ADP spectrum compared to that of the ATP spectrum indicates a small additional monodentate population.

Our scaled ENDOR spectra show that ATP and ADP have almost identical binding affinities to **[Gd.1]**^**+**^, whereas the binding affinity to AMP is approximately 20-fold lower. This is in agreement with the binding affinities previously established via emission-based anion titration experiments with **[Eu.1]**^**+**^(19) and via the EPR line-shape (Figure 5) and relaxation data (Figure S12). Overlaying the Mims ENDOR spectra reveals differences in the precise structure of the spectral envelope, corresponding to differences in couplings resulting from distinct binding modes. Specifically, the line shape of the **[Gd.1]**^**+**^–ADP ENDOR spectrum has more intensity in the central shoulder at ±160 kHz compared with that of **[Gd.1]**^**+**^–ATP, similar to that seen for the AMP ENDOR data. Based on our fittings and simulations shown in Figure 6 and in the SI, this difference is due to ATP adopting only bidentate binding, whereas ADP adopts a mixture of bidentate and monodentate binding to **[Gd.1]**^**+**^. This is again consistent with EXAFS data on **[Eu.1]**^**+**^.

### Determining Coupling Parameters from ^31^P Mims ENDOR

^31^P Mims ENDOR was used to determine the isotropic and dipolar interactions between the Gd(III) center of **[Gd.1]**^**+**^ and the ^31^P atoms of the anionic guests. The best fits to the summed τ-dependent ENDOR spectra are shown in Figure 6 and were simulated using the optimized parameters detailed in Table 4. The contribution of a monodentate or bidentate binding mode was assessed by fitting either one or two ^31^P couplings (*A*_iso_, *A*_dip_, and rhombicity parameter λ, as discussed in the methods section) to the experimental data. The full set of simultaneously fit τ-dependent simulations is shown in Figures S16–S18.

**Table 4:** ENDOR Simulation Fitting Parameters Used to Obtain the Fits Presented in Figure 6^a^

	*A*_iso_(1) (kHz)	*A*_iso_(2) (kHz)	*A*_dip_(1) (kHz)	*A*_dip_(2) (kHz)	λ(1) (kHz)	λ(2) (z)	line width (kHz)	std. (to norm)
AMP 1 × ^31^P	347		661 (3.65 Å)		154		47	2.6 × 10^–3^
AMP 2 × ^31^P	388	322	658 (3.65 Å)	647 (3.67 Å)	142	159	32	2.6 × 10^–3^
ADP 1 × ^31^P	132		690 (3.59 Å)		14		113	6.8 × 10^–4^
ADP 2 × ^31^P	129	131	635 (3.70 Å)	743 (3.50 Å)	11	12	92	5.7 × 10^–4^
ATP 1 × ^31^P	123		689 (3.60 Å)		14		120	6.9 × 10^–4^
ATP 2 × ^31^P	115	127	631 (3.70 Å)	745 (3.50 Å)	5	28	93	5.9 × 10^–4^

a ENDOR simulations were performed using the *EasySpin* program *saffron*.

In simulating the experimental spectra, we found very small differences in dipolar couplings and, thus, Gd^3+^–^31^P distances. These differences are most noticeable in the line shape of the ENDOR spectra measured using short τ values of <0.5 μs and in the line width of longer τ > 1.0 μs spectra. For example, the contribution from a second, slightly more distant nucleus diminishes the height of the shoulders from the *A*_∥_ hyperfine contribution, causing the *A*_⊥_ contribution to appear narrower. Moreover, a larger rhombicity, λ, of the *A*_dip_ component increases the intensity of the central shoulder features of the ENDOR line shape.

### ^31^P ENDOR of **[Gd.1]^+^** with AMP

As compared with the Mims ENDOR spectrum of the ATP and ADP adducts, the **[Gd.1]**^**+**^–AMP Mims ENDOR line shape is visibly narrower and has a more pronounced shoulder feature at approximately ±550 kHz, which is particularly visible in the low-τ-value measurements (Figure S16). This same feature is broadened and is lower in amplitude for the **[Gd.1]**^**+**^–ADP and **[Gd.1]**^**+**^–ATP spectra (Figures S17 and S18). Simulations performed on the AMP-bound **[Gd.1]**^**+**^ complex assuming one ^31^P coupling, fit a rhombic dipolar coupling tensor corresponding to a Gd^3+^–^31^P distance of 3.65 Å and a large isotropic coupling of 347 kHz. Extending the fit to include a second ^31^P resulted in the fit giving two equally weighted and almost identical ^31^P couplings (within 10 kHz), with no improvement in the standard deviation of the fit compared with fitting one ^31^P. This supports the expected monodentate binding of AMP to the **[Gd.1]**^**+**^ complex.

### ^31^P ENDOR of **[Gd.1]^+^** with ADP and ATP

In both the **[Gd.1]**^**+**^–ATP and **[Gd.1]**^**+**^–ADP ENDOR data sets, using two different but equally weighted ^31^P couplings consistently resulted in a slightly improved fit to the experimental data compared to using only one ^31^P coupling. This trend is particularly notable for ATP. This difference is clearly visible when examining the shoulder features at ∼600 kHz in the ENDOR spectra measured using short τ values (0.15–0.3 μs; Figures 6b and S18). In this range, fitting a single ^31^P coupling does not properly account for the broadness of this feature in both data sets, whereas fitting 2 × ^31^P couplings significantly improves the fit, accurately capturing the line shape of the shoulder region. In general, we observed that the fitted line width parameter, as detailed in Table 4, tended to broaden when fitting a single ^31^P to the data set (as seen by the broadening of the central feature in the τ = 2.0 μs simulations in Figures S17 and S18). Here, including the long-τ spectra (τ = 1.0 and 2.0 μs) in the simultaneous fit was valuable because it prevented the simplex algorithm from artificially fitting an oversmoothed/broaden simulation.

The dipolar couplings fitted for each ^31^P nucleus in both the **[Gd.1]**^+^–ADP and **[Gd.1]**^+^–ATP ENDOR data sets are almost the same, and the best fit is achieved with near-axial hyperfine tensors (λ is very small). In each bidentate binding simulation, the distances fit for the two ^31^P nuclei differ only by 0.2 Å, consistent with the distance difference expected based on the DFT models of the possible bidentate modes.^19^ Interestingly, the isotropic coupling fit for all Mims ENDOR simulations is approximately half the magnitude of the isotropic coupling fit for AMP.

Figure 6b shows that while a 2 × ^31^P coupling definitively fits the ATP ENDOR spectrum (in agreement with the EXAFS data suggesting only a bidentate mode), neither the 2 × ^31^P nor the 1 × ^31^P fit captures the small increase in the intensity of the central shoulder features (±160 kHz) of the ADP ENDOR spectrum. We attempted to refine the ENDOR data fitting process based on the EXAFS data indicating that ADP can exhibit both bidentate and monodentate binding to **[Gd.1]**^**+**^. We employed a composite model consisting of the ATP 2 × ^31^P fit and an additional population characterized by the same isotropic and dipolar couplings but a larger rhombicity to match the λ ≈ 120 kHz of the monodentate AMP fit. This performed slightly better in capturing the central shoulders of the ADP data for the higher τ spectra (τ > 1.0 μs) without compromising the fit to the outer regions of the spectrum. While we are confident that these results indicate ADP binds in both a monodentate and bidentate fashion, we cannot confidently determine the relative populations of each without overfitting the data. We can, though, state that the bidentate population must be significantly larger than that of the monodentate population, which is in line with EXAFS results.

## Conclusion

In summary, we have demonstrated that the combination of Eu L_3_-edge EXAFS and high-field EPR/ENDOR spectroscopy can be used in tandem to distinguish the binding geometries of nucleoside phosphate anions ATP, ADP, and AMP to lanthanide complexes. We showed that ATP unambiguously binds to the lanthanide center in a bidentate manner, forming a stable 1:1 host–guest structure. This is consistent with previous solution NMR data, indicating the binding of ATP via the α- and γ-phosphate groups. Crucially, EXAFS analysis allowed us to definitively rule out the possibility of a 2:1 host–guest complex.

The combined use of EXAFS and EPR techniques has revealed that ADP can readily adopt both bidentate and monodentate modes in solution, with a clear preference for the former. Notably, the propensity for ADP to bind in monodentate fashion was not apparent from previous emission and NMR spectral studies, where only the most emissive bidentate **[Eu.1]**^**+**^–ADP species was observed in the emission spectrum, and significant line-broadening was observed in the NMR spectrum. Additionally, we have shown that AMP binds exclusively in a monodentate manner and that its binding affinity is smaller in order of magnitude.

This study highlights the benefits of combining EXAFS and EPR spectroscopy to guide the design of new macrocyclic complexes that undergo specific anion-induced structural changes, thus producing the desired optical response. For example, lanthanide complexes incorporating sterically demanding ligands can now be designed for the selective recognition of AMP over the more highly charged ATP, harnessing the preference for monodentate binding of the former anion.^30^ Developing molecular hosts with a high degree of thermodynamic selectivity between ATP and ADP is potentially more challenging. However, the requirement for flexible pendant arms to enable chelation of the larger ATP molecule, combined with the knowledge that ADP can adopt different binding modes, offers valuable starting points to address this challenge in future lanthanide probe design.

## Experimental Section

### General Considerations

Phosphate anions adenosine 5-diphosphate monosodium salt and adenosine 5-triphosphate disodium hydrate were purchased from Carbosynth. **[Eu.1]**^**+**^ was synthesized, purified, and characterized according to the previously reported procedure.^31^

### EXAFS Sample Preparation

For XAS measurements, a solid sample of **[Eu.1]**^**+**^ (approximately 18 mg) was compressed into a pellet and securely enclosed with Kapton tape. For solution samples, **[Eu.1]**^**+**^ (1.8 mg) was dissolved in 10 mM HEPES buffer (0.2 mL) at pH 7.0 to give a 10 mM solution and sealed with Kapton tape. For solutions involving anions, stock solutions of ADP or ATP in 10 mM HEPES buffer were added to a solution of **[Eu.1]**^**+**^ to give a final 1:1 molar ratio. For measurements at 77 K, samples were rapidly frozen in liquid nitrogen while still encased in the Kapton tape and then placed in the cryogenic chamber. The sample names, compositions, and temperatures are given in Table S1.

### XAS Measurement

X-ray absorption spectroscopy (XAS) data for **[Eu.1]**^**+**^ were obtained at Beamline I20 at Diamond Light Source, Oxford, U.K. Beamline I20 offers an X-ray energy range of 4.5–20 keV, with a resolution of Δ*E*/*E* = 1.3 × 10^–4^ with an unfocused beam size of 400 × 300 μm, utilizing a Si(111) monochromator consisting of a cryo-cooled first crystal. A harmonic rejection mirror was put in place for all experiments. For the XAS scans focused on the L-edge, a typical scan requires a range of 50–200 eV before the absorption edge for the preedge fit and 100–1000 eV above the absorption edge for the postedge fit. In our specific case, XAS scans for the L-edge utilized a range of 100 eV prior to the absorption edge and 500 eV following the absorption edge. Figure S4 shows the experimental setup for XAS measurements in transmission mode at Beamline I20. The EXAFS data for all Eu samples were collected at the Eu L_3_-edge (6977 eV) using the transmission mode at both room temperature (293 K) and low temperature (77 K). All spectra were referenced against an iron foil (7112 eV).

### EXAFS Data Processing

XAFS data processing was performed using IFEEFIT with the Demeta package (Athena and Artimus). *S*_0_^2^ values for all analyses was set at 1. Given the number of paths and conformational flexibility of the complexes, particularly in solution, fitting was performed using predefined models with fixed coordination numbers (CN). The parameters investigated were *E*_0_, the path length, and the 2σ^2^ value (which reflects the structural and vibrational disorder of the system). The quality of fit and physical realism of modeled paths were used to determine which model most accurately reflected the specific samples. Fitting with floated CN values produced acceptable fits, but the notable error in determined values limits the use of this methodology in determining binding modes of ATP and ADP to the Eu(III) center. While the Eu–C paths are required for the fit, these paths will not be discussed, and the data will be presented in the SI. This is because the fit of these paths is oversimplified for the complex and as they are not directly coordinated to the europium ion, little meaningful structural information can be determined from them. Comparison of fits and data in *k*-, *R*-, and *q*-space are shown in Figures S5–S11.

#### Model 1 (monodentate)

 was developed from a structural model determined from the previously reported X-ray crystal structure of the monoaqua europium complex (Figure S3).^21^ A single Eu–O path was fitted with a CN of 3, simplifying the coordination geometry from that observed by XRD of two degenerate Eu–O paths associated with the carboxylate arms at 2.322 Å and another Eu–O associated with bound water at 2.388 Å. Regarding Eu–N coordination, two distinct path lengths were fitted: one for the four nitrogen atoms within the macrocyclic ring and another for the two quinoline nitrogen atoms. Additionally, three Eu–C distances, comprising of two with a count of 8 and one with a count of 2, were fitted using a single expansion factor. The combination of path amalgamation and fixed coordination numbers enabled fits to be performed with a viable number of variables.

#### Model 2 (bidentate)

 was developed based on results determined by previous ^31^P NMR spectroscopy results. In this EXAFS model, the Eu–O coordination number was increased from 3 to 4 to reflect bidentate binding. Eu–N paths associated with the macrocyclic ring were retained as in model 1 with a CN of 4. Attempts were made to reduce the Eu–N associated with quinoline nitrogen to reflect one arm being displaced by ATP or ADP with fits using a CN of 1 or the total removal of these paths being used. Eu–C distances and CN were fitted using the same parameters as model 1.

### EPR Characterization

For EPR analysis, the gadolinium complex **[Gd.1]**^**+**^ of the same ligand was used. Gd(III) is an *S* = ^7^/_2_ spin system with an isotropic **g**-tensor. The Gd(III) EPR spectrum is characterized by an intense, narrow line corresponding to the −^1^/_2_ ↔ ^1^/_2_ transition of the spin manifold, whereas higher spin transitions manifest as a broad, low-intensity envelope across ∼200 mT. The narrow line shape for the −^1^/_2_ ↔ ^1^/_2_ transition means that the entire transition intensity can be easily excited with a short microwave pulse at a single field position. The isotropy of the Gd(III) **g**-tensor also further simplifies the measurement by eliminating the need for orientation-selection considerations or measuring at multiple field positions. ^31^P, which is an *I* = ^1^/_2_ nucleus, can be detected indirectly via the electron spin using double resonance spectroscopy techniques such as ENDOR, which probe the isotropic and dipolar coupling between the Gd(III) electrons and the ^31^P nuclei of the anions.

Stock solutions of **[Gd.1]**^**+**^ were prepared in a 75% methanol-*d*_4_/25% D_2_O solvent mixture, while AMP, ADP, and ATP stocks were prepared in D_2_O. The final W-band EPR samples were prepared in 1:10 **[Gd.1]**^**+**^ to nucleoside phosphate anion molar concentration ratios, to final **[Gd.1]**^**+**^ concentrations of 150 μM diluted with D_2_O and 10% glycerol-*d*_8_ as the cryoprotectant. Samples were transferred to quartz W-band capillaries in 4 μL aliquots (1.6 mm O.D. and 1.0 mm I.D.) and stored at −80 °C. The samples were prefrozen in liquid nitrogen before being inserted into the cryostat and W-band resonator.

EPR measurements were performed at 10 K on a modified W-band 94 GHz E680 Bruker spectrometer^32^ equipped with a TE_011_-mode EPR/ENDOR resonator in a helium gas flow cryostat (Oxford Instruments). The EPR field-sweep line-shape measurements were recorded using a two-pulse Hahn echo sequence π/2−τ–π–τ–echo, with π/2 = 20 ns, π = 40 ns, and τ = 300 ns. *T*_M_ relaxation was measured by recording the decay of the integrated echo intensity with time, using the pulse sequence π/2–(*t* + d*t*)–π–(*t* + 2d*t*)–echo, with the same pulse lengths and timings as above. The electronic *T*_1_ relaxation was measured by the inversion–recovery sequence π–(*t* + d*t*)–π/2−τ–π–echo, recording the integrated echo intensity as a function of the delay time *t* (incremented with the time step dt), using π/2 = 40 ns, π = 80 ns, and τ = 300 ns.

### ^31^P ENDOR Measurements and Simulations

The ^31^P Mims ENDOR spectra were measured using the standard pulse sequence π/2−τ–π/2–*t*_π,RF_–π/2−τ–echo, with π/2 = 12 ns and *t*_π,RF_ = 41 μs. To properly sample the ENDOR line shape, the spectra were measured at different τ values varied (τ = 0.15, 0.22, 0.3, 0.5, 1.0, or 2.0 μs) to overcome “blindspotting” artifacts that form as a result of the spin dynamics in the Mims pulse sequence.^28^ For the **[Gd.1]**^**+**^–AMP complex, τ values below 0.22 μs could not be used as the signal-to-noise was too low due to the lower AMP binding affinity. The RF power (source 100 W) was adjusted to achieve the maximum ENDOR response. The attenuation used was 0 dB and was determined by microwave nutation, using the proton line for calibration of the RF pulse settings and the ^31^P resonance frequency at the set magnetic field strength. The ENDOR spectra were acquired in stochastic mode, with 1 shot per (sample) point and a 4 ms shot repetition time system. The accumulation time was about 1.5 h per τ-value per spectrum.

The ENDOR intensities were scaled to the Gd(III) echo intensity to compare the anion binding affinities to **[Gd.1]**^**+**^. To do this, the ^31^P ENDOR intensity was plotted as a percentage of the EPR echo intensity by dividing by the ^31^P ENDOR baseline value, where a ^31^P signal was absent. The spectra were then symmetrized about the central ^31^P frequency corresponding to the given magnetic field position.

The experimental ^31^P ENDOR spectra were modeled by fitting the Gd(III) ^31^P hyperfine interaction, characterized by the hyperfine tensor:
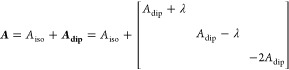
where *A*_iso_ is the isotropic hyperfine interaction resulting from orbital overlap, *A*_dip_ is the dipolar coupling interaction, which is distance-dependent, and the dipolar coupling frequency ω_d_ is given by
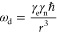
λ introduces rhombicity into the otherwise axial dipolar coupling tensor.

To simulate the experimental ^31^P Mims ENDOR spectra, the *EasySpin* program *saffron* was used with a simplex minimization fitting procedure. Spectra for the same sample measured at different τ values were fit simultaneously so that one set of coupling parameters would apply to all measurements performed on that sample. In this fitting procedure, the ^31^P coupling terms *A*_iso_, *A*_dip_, and λ and the Gaussian ENDOR line width (Sys.lwEndor) were varied until the sum of the standard deviations of the experimental and simulated spectra was minimized (convergence criteria 10^–4^ root-mean-square deviation). Either one ^31^P coupling or two equally weighted ^31^P couplings were fit to determine whether a monodentate or bidentate mode of the anion to the **[Gd.1]**^**+**^ complex was more likely.

## Supporting Information Available

Illustrations of anion binding modes of **[Eu.1]**^**+**^ derived from EXAFS and EPR data, emission spectral changes in the presence of selected anions, and XAFS measurements of **[Eu.1]**^**+**^ in the presence and absence of anions, including EXAFS fit plots and plotting parameters, EPR relaxation measurements, line-broadening analyses, and ^31^P ENDOR data (PDF)

## Abbreviations

ADP: adenosine diphosphate
AMP: adenosine monophosphate
ATP: adenosine triphosphate
CN: coordination number
DFT: density functional theory
ENDOR: electron nuclear double resonance
EPR: electron paramagnetic resonance
EXAFS: extended X-ray absorption fine structure
GHz: gigahertz
HEPES: 4-(2-hydroxyethyl)-1-piperazineethanesulfonic acid
I.D.: inner diameter
MHz: megahertz
NMR: nuclear magnetic resonance
O.D.: outer diameter
RF: radio frequency
XAFS: X-ray absorption fine structure
XAS: X-ray absorption spectroscopy
XRD: X-ray diffraction
ZFS: zero-field splitting
