# Supplementary material for: Elucidating Polyphosphate Anion Binding to Lanthanide Complexes Using EXAFS and Pulsed EPR Spectroscopy
Source: Inorg Chem. 2024 Oct 16;63(43):20726–36. doi: 10.1021/acs.inorgchem.4c03399 (PMC11523222; doi:10.1021/acs.inorgchem.4c03399)
Supplement: Supplementary file 1 — ic4c03399_si_001.pdf [file ic4c03399_si_001.pdf]

Supporting Information

Elucidating Polyphosphate Anion Binding to Lanthanide Complexes using EXAFS and Pulse EPR Spectroscopy

Hannah K. Pyle,[a] Martyna Judd,[b] Anthony Barancewicz,[b] Alexander J. Mayer,[a] Nicholas Cox*,[b] Simon A. Kondrat*[a] and Stephen J. Butler*[a]

[a] Department of Chemistry, Loughborough University, Epinal Way, Loughborough LE11 3TU, UK.
[b] Research School of Chemistry, The Australian National University, Canberra, ACT 2605, Australia.
Corresponding Author Email: S.J.Butler@lboro.ac.uk

Contents

Anion Binding Modes and Emission Spectra of [**Eu.1**]$^+$ S2
X-ray Structure and HPLC trace of [**Eu.1**]$^+$ S3
XAS measurement S4
EXFAS Measurements of [**Eu.1**]$^+$ in the Absence and Presence of Anions S5
EPR Relaxation Measurements S12
EPR Line Broadening. S13
Complete τ-dependent ^{31}P ENDOR Fits S16
References S18

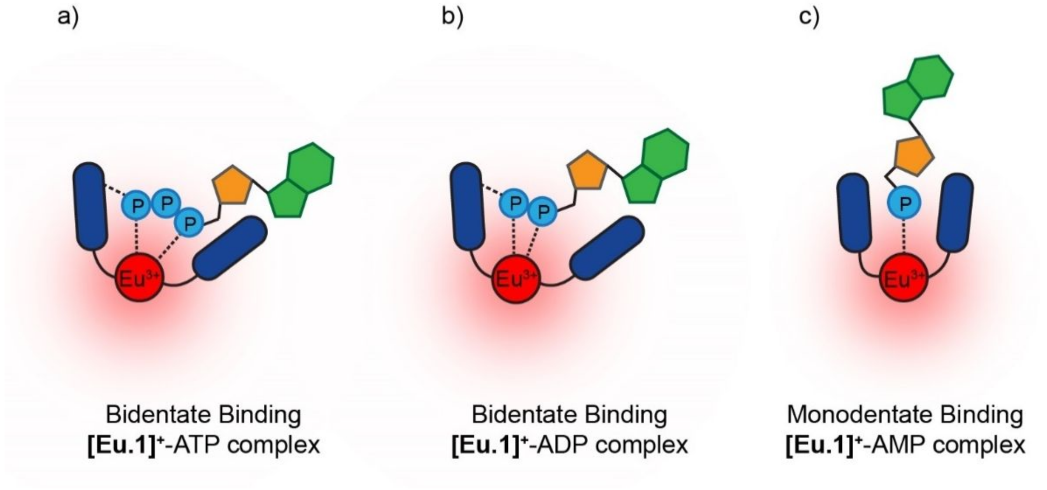

Figure S1. Simplified illustrations of the anion binding modes of **[Eu.1]**$^+$ derived from EXAFS and EPR data, depicting a) ATP binding in a bidentate manner via the α- and γ-phosphates; b) ADP binding in a bidentate manner via the α- and β-phosphates, and c) AMP binding in a monodentate manner.

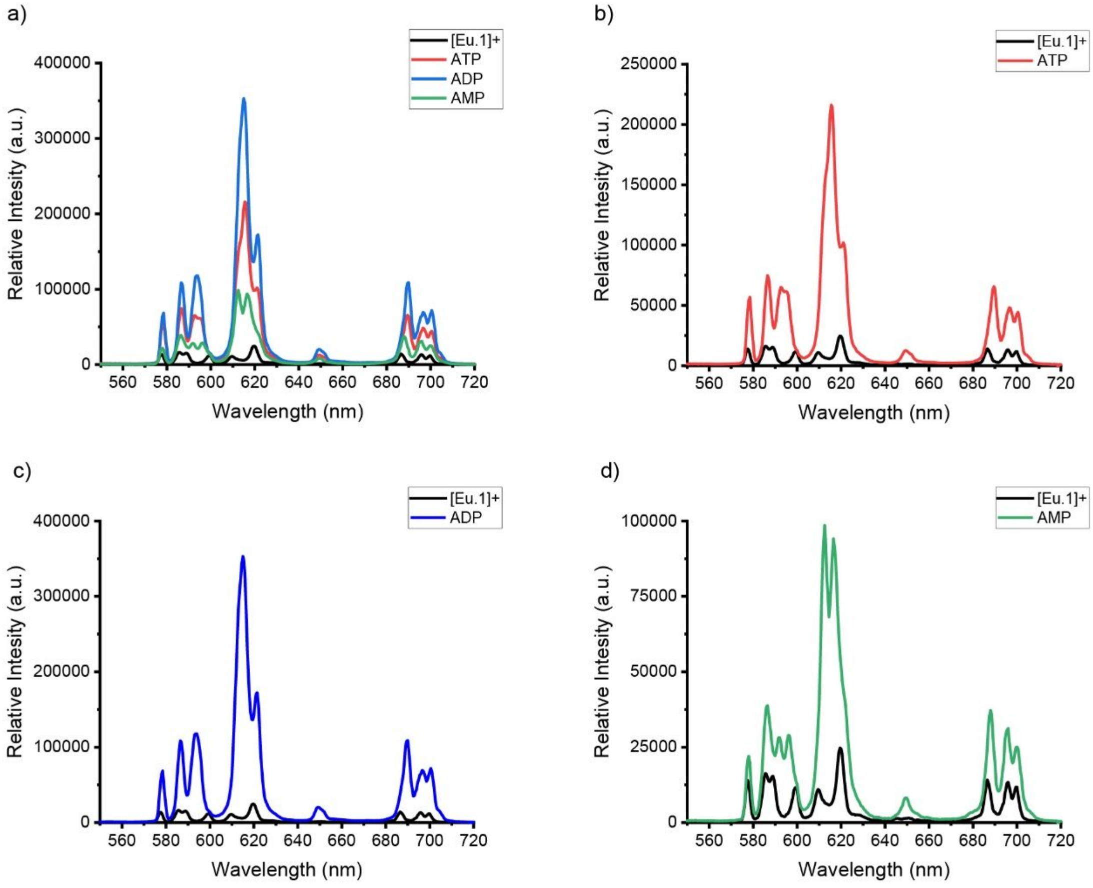

Figure S2. Change in emission spectra of **[Eu.1]**$^+$ (black) in the presence of 1 mM ATP (red), ADP (blue), and AMP (green). Notably, the emission spectral profile of **[Eu.1]**$^+$ is very similar in the presence of ATP and ADP, whereas AMP induces a distinctive splitting pattern within the ΔJ=1 (582–605 nm) and ΔJ=2 (605–630 nm)

emission bands. Panel a) shows the full emission spectra for ATP, ADP, and AMP, with focused views for b) ATP, c) ADP, and d) AMP.

X-ray Structure and HPLC Trace of [Eu.1]+

The synthesis and full characterisation of [**Eu.1**]+ is reported previously, including ^{1}H NMR spectra, ESI high resolution mass spectra, analytical HPLC chromatogram, and X-ray crystal structure.[1, 2] For convenience, the X--ray crystal structure of the ternary adduct of [**Eu.1**]+ and formate is provided in Figure S3 below, together with the analytical RP-HPLC trace of [**Eu.1**]+.

a)

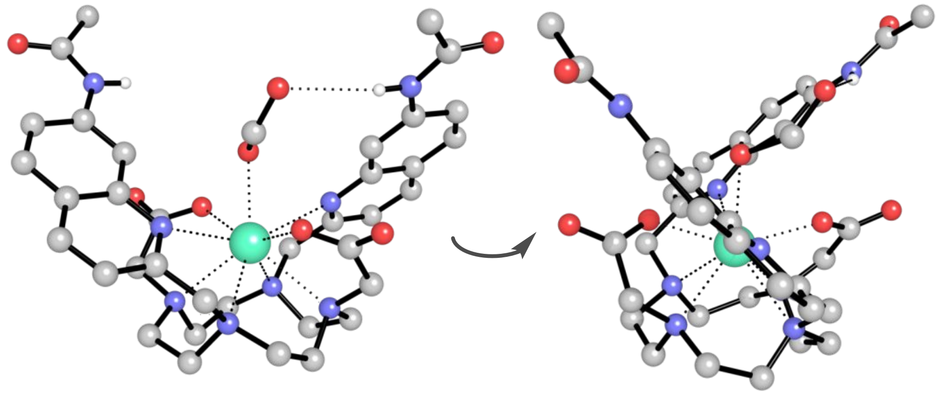

b)

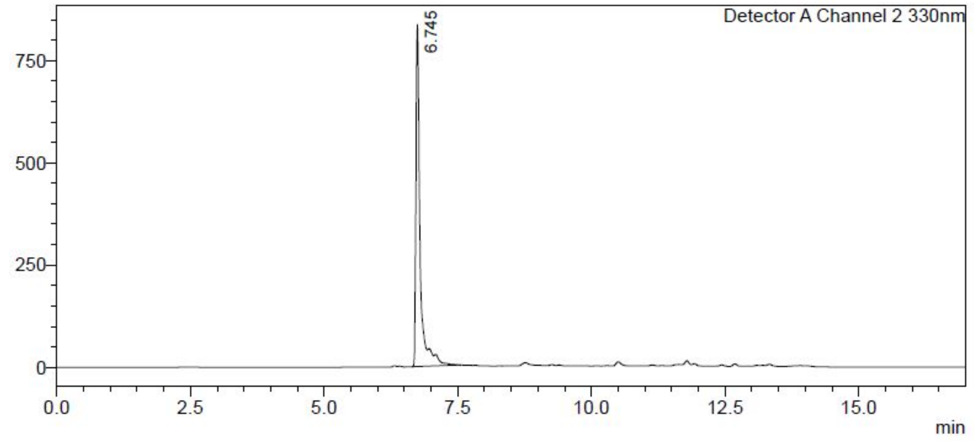

Figure S3. a) Two views of the X-ray crystal structure of the ternary adduct of **[Eu.1]+** and formate, crystallised from acetonitrile/water (1:1). Key: Eu turquoise, C grey, N blue, O red, H white. Most H atoms and 7 water molecules of crystallisation are omitted for clarity. Reproduced from Ref. 1 with permission from the Royal Society of Chemistry. **b)** Analytical RP-HPLC trace of [**Eu.1**]+; t_R = 6.75 min [gradient: 2 – 100% acetonitrile in 25 mM NH_4CO_3 over 15 min]. Analytical RP-HPLC was performed using a XBridge C18 5 µm 4.6 × 100 mm

at a flow rate maintained at 2.0 mL min^{-1}. Reproduced from Ref. 2 with permission from the Royal Society of Chemistry.

Table S1. Samples used in XAS measurements, including composition and temperature.

Sample	Sample Name	State	[EuL2] (mM)	[anion] (mM)	Temperature (K)
1	**[Eu.1]**+ Solid	Solid	10	-	293 K
2	**[Eu.1]**+ 77 K	Liquid	10	-	77 K
3	**[Eu.1]**+ 293 K	Liquid	10	-	293 K
4	**[Eu.1]**+-ATP 77 K	Liquid	10	10	77 K
5	**[Eu.1]**+-ATP 293 K	Liquid	10	10	293 K
6	**[Eu.1]**+-ADP 77 K	Liquid	10	10	77 K
7	**[Eu.1]**+-ADP 293 K	Liquid	10	10	293 K

XAS Measurement

The X-ray absorption spectroscopy (XAS) data for **[Eu.1]**+ was obtained at beamline I20 located at Diamond Light Source in Oxford, UK. Beamline I20 offers an X-ray energy range of 4.5 - 20 keV, with a resolution of $\Delta E/E=1.3x10^{-4}$ with an unfocused beam size of 400 x 300 μm, utilizing a Si (111) monochromator consisting of a cryo-cooled first crystal.

For the XAS scans focused on the L-edge, a typical scan requires a range of 50–200 eV before the absorption edge for the pre-edge fit and 100–1000 eV above the absorption edge for the post-edge fit. In our specific case, XAS scans for the L-edge utilized a range of 100 eV prior to the absorption edge and 500 eV following the absorption edge.

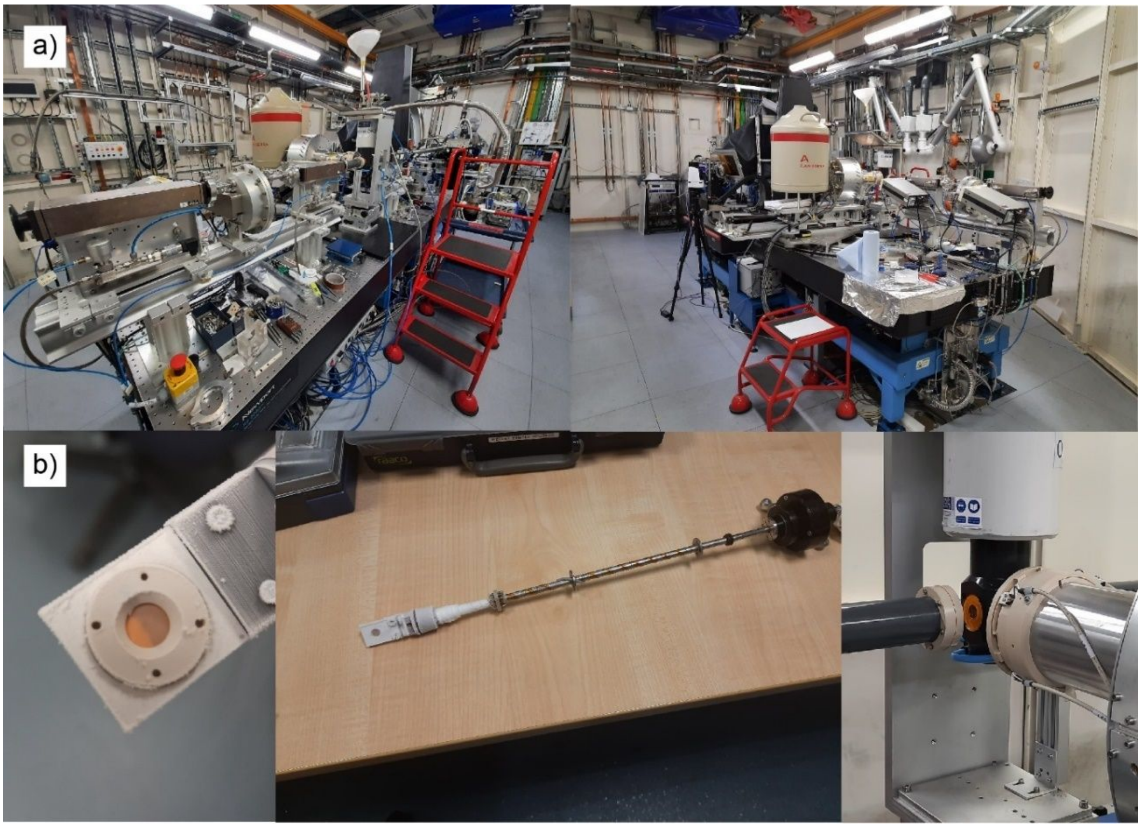

Figure S4. Images of the experimental setup for XAS measurements in transmission mode at the I20 beamline. Panel a) shows a broad view of the setup, while panel b) offers a close-up of the sample, illustrating its loading

and the sample chamber. The EXAFS data for all Eu(III) samples were collected at the Eu L^3-edge (6,977 eV) using the transmission mode at both room temperature (293 K) and low temperature (77 K).

EXAFS Measurements of [Eu.1]+ in the Absence and Presence of Anions

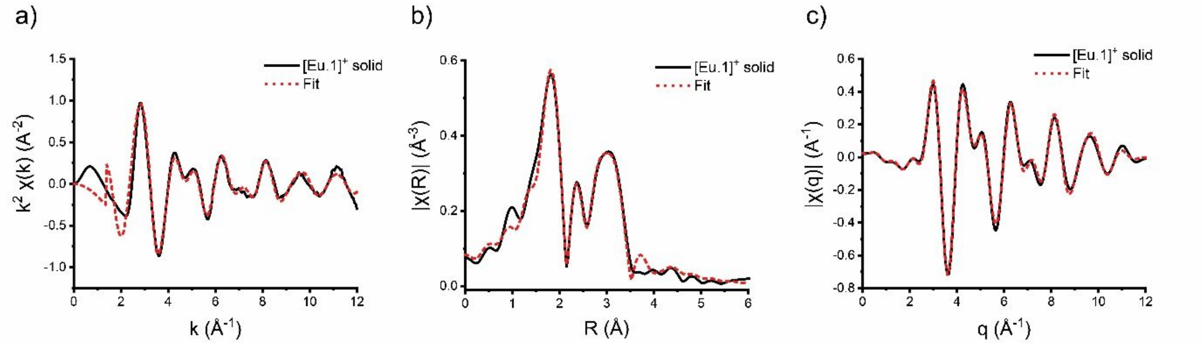

Figure S5. Comparison of solid **[Eu.1]+** data and fits using model 1 in; a) k^2-weighted space; b) k^2-weighted R-; c) EXAFS data in q-space.

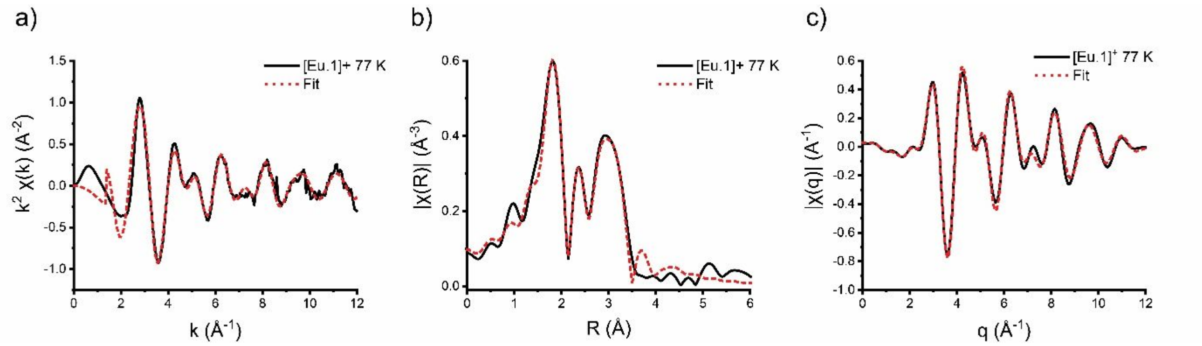

Figure S6. Comparison of **[Eu.1]+** dissolved in 10 mM HEPES buffer solution (pH 7.0) and flash frozen at 7 7k data and fits using model 1 in; a) k^2-weighted space; b) k^2-weighted R-; c) EXAFS data in q-space.

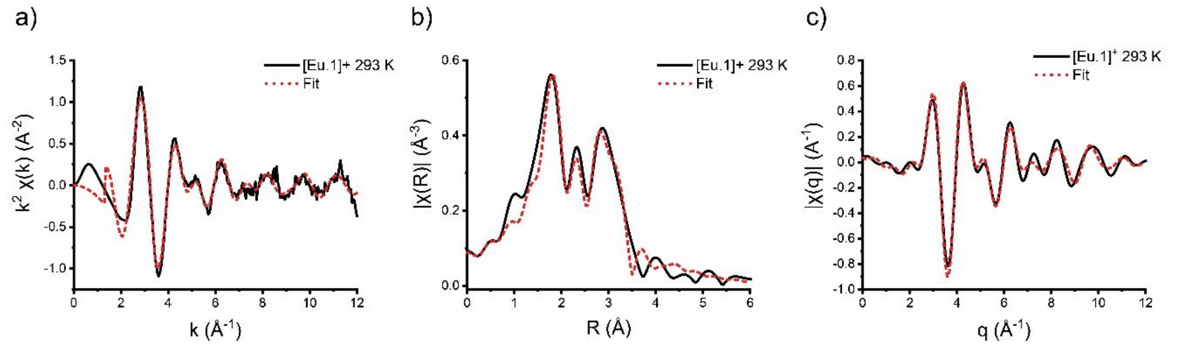

Figure S7. Comparison of **[Eu.1]+** dissolved in 10 mM HEPES buffer solution (pH 7.0) at 293 K data and fits using model 1, in; a) k^2-weighted space; b) k^2-weighted R-; c) EXAFS data in q-space.

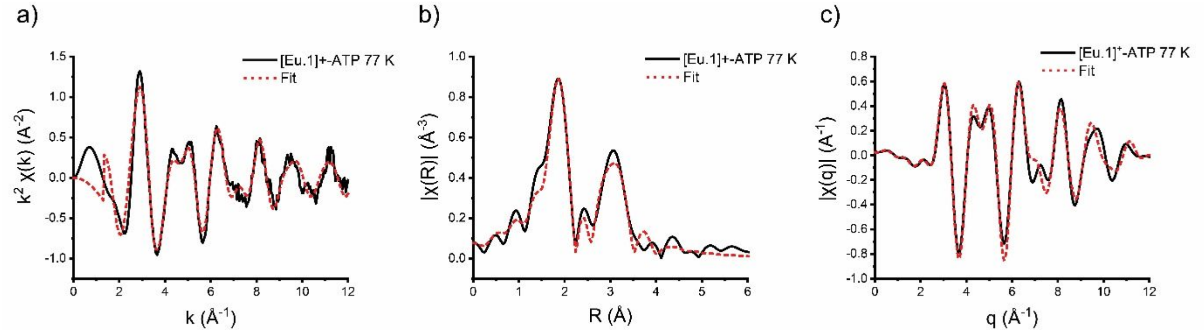

Figure S8. Comparison of ATP bound to **[Eu.1]**$^+$ data in a 1:1 molar ratio, measured in 10 mM HEPES buffer solution (pH 7.0) at 77 K and fits using model 2 in; a) k^2-weighted space; b) k^2-weighted R-; c) EXAFS data in *q*-space.

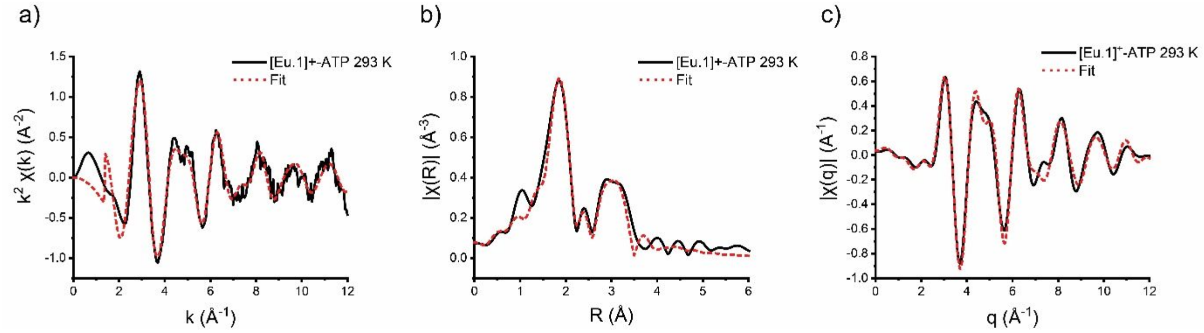

Figure S9. Comparison of ATP bound to **[Eu.1]**$^+$ data in a 1:1 molar ratio, measured in 10 mM HEPES buffer solution (pH 7.0) at 293 K and fits using model 2 in; a) k^2-weighted space; b) k^2-weighted R-; c) EXAFS data in *q*-space.

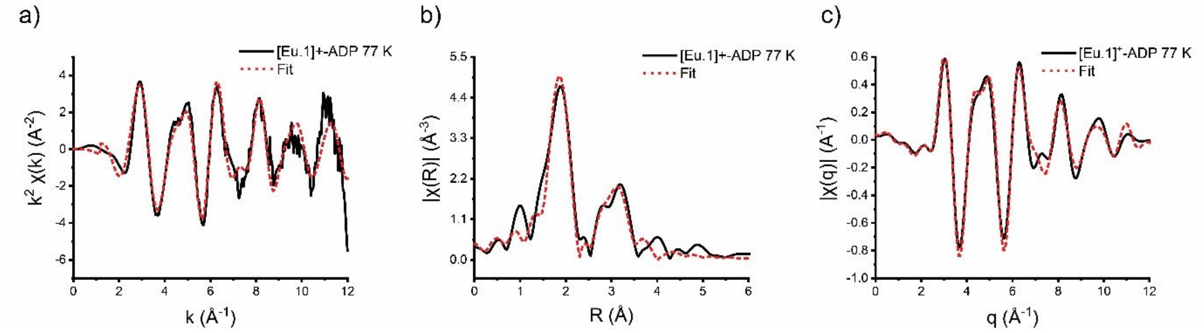

Figure S10. Comparison of ADP bound to **[Eu.1]**$^+$ data in a 1:1 molar ratio, measured in 10 mM HEPES buffer solution (pH 7.0) at 77 K and fits using model 2 in; a) k^2-weighted space; b) k^2-weighted R-; c) EXAFS data in *q*-space.

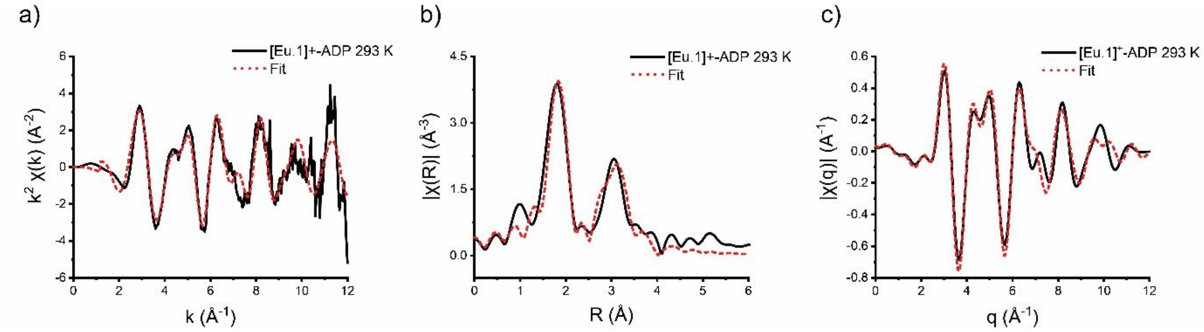

Figure S11. Comparison of ADP bound to $[Eu.1]^+$ data in a 1:1 molar ratio, measured in 10 mM HEPES buffer solution (pH 7.0) at 293 K and fits using model 2 in; a) k^2-weighted space; b) k^2-weighted R-; c) EXAFS data in *q*-space.

Table S2. EXAFS plotting parameters for **[Eu.1]+** measured as solid and aqueous solutions, at 77 K and 293 K.

Sample	Path	*N*[a]	R [Å][b]	$2\sigma^2$ [Å^2][b]	E_f [eV][b]	R_{factor} [χ^2]
**Solid [Eu.1]+**	Eu-O	3	2.38(1)	0.0021(1)	1.4 (4)	0.014 [326]
	Eu-O (CN float)	3.1(5)	2.38(1)	0.0021[c]		
	Eu-N(1)	4	2.61(1)	0.006(4)		
	Eu-N(2)	2	2.78(4)	0.006[c]		
	Eu-C(1)	8	3.50(4)[d]	0.008(1)[e]		
	Eu-C(2)	2	3.35(4)[d]	0.008(1)[e]		
	Eu-C(3)	8	3.96(4)[d]	0.008(1)[e]		
**[Eu.1]+ 77 K**	Eu-O	3	2.39(1)	0.0022(1)	7(1)	0.019 [72]
	Eu-N(1)	4	2.61(2)	0.006(7)		
	Eu-N(2)	2	2.77(7)	0.0064[c]		
	Eu-C(1)	8	3.50(5)[d]	0.007(1)[e]		
	Eu-C(2)	2	3.34(5)[d]	0.007(1)[e]		
	Eu-C(3)	8	3.94(5)[d]	0.007(1)[e]		
**[Eu.1]+ 293 K**	Eu-O	3	2.40(2)	0.004(1)	7(1)	0.040 [78]
	Eu-N(1)	4	2.63(2)	0.006[c]		
	Eu-N(2)	2	2.82(5)	0.007[c]		
	Eu-C(1)	8	3.49(6)[d]	0.008(2)[e]		
	Eu-C(2)	2	3.34(6)[d]	0.008(2)[e]		
	Eu-C(3)	8	3.94(6)[d]	0.008(2)[e]		

[a] *N*– coordination number. [b] Uncertainty in the interatomic distances, Debye-Waller factors and E0 values is given in brackets for the last decimal place.[c] path defined at Eu-N1 x 1.1 during fit. [d] fitted using a single expansion parameter [e] all Eu-C fitted using the same $2\sigma^2$. S_0^2 set at 1. *K* fitting range of 3-11 Å^{-1} and R fitting range of 1-4 Å.

Table S3. EXAFS plotting parameters for **[Eu.1]+**–ATP data at 77 K, and **[Eu.1]+**–ADP data at 77 K. Model 1 utilises **[Eu.1]+** solid fitting parameters, whereas model 2 represents a bidentate anion binding model.

Sample	Path	*N*[a]	R [Å][b]	$2\sigma^2$ [$Å^2$][b]	E_f [eV][b]	R_{factor} [χ^2]
**[Eu.1]+**–ATP Model 1	Eu-O	3	2.39(1)	0.001(1)	8(3)	0.059 [255]
	Eu-N(1)	4	2.58(78)	0.02(10)		
	Eu-N(2)	2	2 (2)	0.021c		
	Eu-C(1)	8	3.51(1)d	0.004(2) e		
	Eu-C(2)	2	3.35(1) d	0.004(2) e		
	Eu-C(3)	8	3.95(1) d	0.004(2) e		
**[Eu.1]+** –ATP Model 2 with N2	Eu-O	3	2.38(1)	0.0022(1)	7(1)	0.049 [227]
	Eu-N(1)	4	2.61(20)	0.016		
	Eu-N(2)	2	2.64(70)	0.017c		
	Eu-C(1)	8	3.50(5)d	0.004(2) e		
	Eu-C(2)	2	3.34(5) d	0.004(2) e		
	Eu-C(3)	8	3.94(5) d	0.004(2) e		
**[Eu.1]+** –ATP Model 2 without N2	Eu-O	3	2.40(2)	0.004(1)	7(1)	0.040 [78]
	Eu-N(1)	4	2.63(2)	0.006c		
	Eu-C(1)	8	3.50(2)d	0.005(1)e		
	Eu-C(2)	2	3.34(2) d	0.005(1) e		
	Eu-C(3)	8	3.94(2) d	0.005(1) e		
**[Eu.1]+** –ADP Model 1	Eu-O	3	2.31(1)	0.0006(27)	5(1)	0.027 [159]
	Eu-N(1)	4	2.47(3)	-0.001(3)		
	Eu-N(2)	2	2.65(3)	-0.0012 c		
	Eu-C(1)	8	3.48(6)d	0.007(2) e		
	Eu-C(2)	2	3.33(6)d	0.007(2) e		
	Eu-C(3)	8	3.93(6)d	0.007(2) e		
**[Eu.1]+** –ADP Model 2 without N2	Eu-O	4	2.36(2)	0.0038(4)	5(2)	0.034 [199]
	Eu-N(1)	4	2.66(6)	0.007(16)		
	Eu-C(1)	8	3.48(1) d	0.006(2) e		
	Eu-C(2)	2	3.32(1) d	0.006(2) e		
	Eu-C(3)	8	3.92(1) d	0.006(2) e		

[a] *N*– coordination number. [b] Uncertainty in the interatomic distances, Debye-Waller factors and E0 values is given in brackets for the last decimal place.[c] path defined at Eu-N1 x 1.1 during fit. [d] fitted using a single expansion parameter [e] all Eu-C fitted using the same $2\sigma^2$. S_0^2 set at 1. *K* fitting range of 3-11 $Å^{-1}$ and R fitting range of 1-4 Å.

Table S4. EXAFS plotting parameters for **[Eu.1]**⁺–ATP EXAFS data at 293 K and **[Eu.1]**⁺–ADP EXAFS data at 293 K. Model 1 utilises **[Eu.1]**⁺ solid fitting parameters, whereas model 2 represents a bidentate anion binding model.

Sample	Path	*N*[a]	R [Å][b]	$2\sigma^2$ [$Å^2$][b]	E_f [eV][b]	R_{factor} [χ^2]
**[Eu.1]**⁺-ATP Model 1	Eu-O	3	2.34(2)	0.0002(32)	7(2)	0.036 [81]
	Eu-N(1)	4	2.51(3)	0.00024(52)		
	Eu-N(2)	2	2.70(5)	0.00027 c		
	Eu-C(1)	8	3.52(1)d	0.008 (4) e		
	Eu-C(2)	2	3.36(1) d	0.008 (4) e		
	Eu-C(3)	8	3.97(1) d	0.008 (4) e		
**[Eu.1]**⁺-ATP Model 2	Eu-O	4	2.38(2)	0.003(1)	7(2)	0.039 [88]
	Eu-N(1)	4	2.59(3)	0.008(4)		
	Eu-C(1)	8	3.48(1)d	0.007 (2) e		
	Eu-C(2)	2	3.33(1) d	0.007 (2) e		
	Eu-C(3)	8	3.92(1) d	0.007 (2) e		
**[Eu.1]**⁺-ADP Model 1	Eu-O	3	2.33	0.0002(14)	6(1)	0.036 [57]
	Eu-N(1)	4	2.52	0.0002(20)		
	Eu-N(2)	2	2.73	0.00027c		
	Eu-C(1)	8	3.48(8)d	0.006 (2) e		
	Eu-C(2)	2	3.33(8) d	0.006 (2) e		
	Eu-C(3)	8	3.92 (8) d	0.006 (2) e		
**[Eu.1]**⁺-ADP Model 2	Eu-O	4	2.36(2)	0.004(1)	8(2)	0.050 [75]
	Eu-N(1)	4	2.59(4)	0.0010(5)		
	Eu-C(1)	8	3.48(8)d	0.006 (2) e		
	Eu-C(2)	2	3.33(8) d	0.006 (2) e		
	Eu-C(3)	8	3.92 (8) d	0.006 (2) e		

[a] *N*– coordination number. [b] Uncertainty in the interatomic distances, Debye-Waller factors and E0 values is given in brackets for the last decimal place.[c] path defined at Eu-N1 x 1.1 during fit. [d] fitted using a single

expansion parameter [e] all Eu-C fitted using the same $2\sigma^2$. S_0^2 set at 1. *K* fitting range of 3-11 $Å^{-1}$ and R fitting range of 1-4 Å.

Table S5. EXAFS plotting parameters for **[Eu.1]⁺** frozen sample measured at 77 K. The table demonstrates the unsatisfactory effect of adding an Eu-P path, where overlapping carbon scatterer paths allowed Eu-P to be fitted even in the absence of ATP or ADP.

Sample	Path	*N*[a]	R [Å][b]	$2\sigma^2$ [$Å^2$][b]	E_f [eV][b]	R_{factor} [χ^2]
**[Eu.1]⁺** 77 K	Eu-O	3	2.39(9)	0.0014(12)	3.1(9)	0.006 [29]
	Eu-N(1)	4	2.61(15)	0.0040(37)		
	Eu-N(2)	2	2.77(4)	0.0042[c]		
	Eu-C(1)	8	3.48[d]	0.0066(12)[e]		
	Eu-C(2)	2	3.25[d]	0.0048(47)[e]		
	Eu-C(3)	8	3.84[d]	0.0072[e]		
	Eu-P	1	3.77(4)	0.0071		

[a] *N* – coordination number. [b] Uncertainty in the interatomic distances, Debye-Waller factors and E0 values is given in brackets for the last decimal place.[c] path defined at Eu-N1 x 1.1 during fit. [d] fitted using a single expansion parameter [e] all Eu-C fitted using the same $2\sigma^2$. S_0^2 set at 1. *K* fitting range of 3-11 $Å^{-1}$ and R fitting range of 1-4 Å.

EPR Supplementary Information

EPR Relaxation Measurements

The T_1 and T_2 relaxation data was measured as described in the methods paper of the main text. The experimental relaxation measurements and corresponding fits are shown in Fig. S12. The fitting parameters are tabulated in Table S5.

Table S6. Parameters used to simulate the relaxation data of the bound **[Gd.1]⁺** complex. A biexponential function $-A_1[\exp(-x/\tau_1)+A_2\exp(-x/\tau_2)]$ was used to fit the T_1 relaxation, and a monoexponential decay function $\exp(-x/\tau)$was used to fit the T_2relaxation.

	T_1 - τ_1 (μs)	T_1 - τ_2 (μs)	*1-1/e*	T_2 *(μs)*	*1/e (μs)*
**[Gd.1]⁺**–AMP	89.34	21.3	39	15.5	6.96
**[Gd.1]⁺**–ADP	85.7	19.6	36	14.3	6.67
**[Gd.1]⁺**–ATP	81.0	18.0	32	12.9	5.9

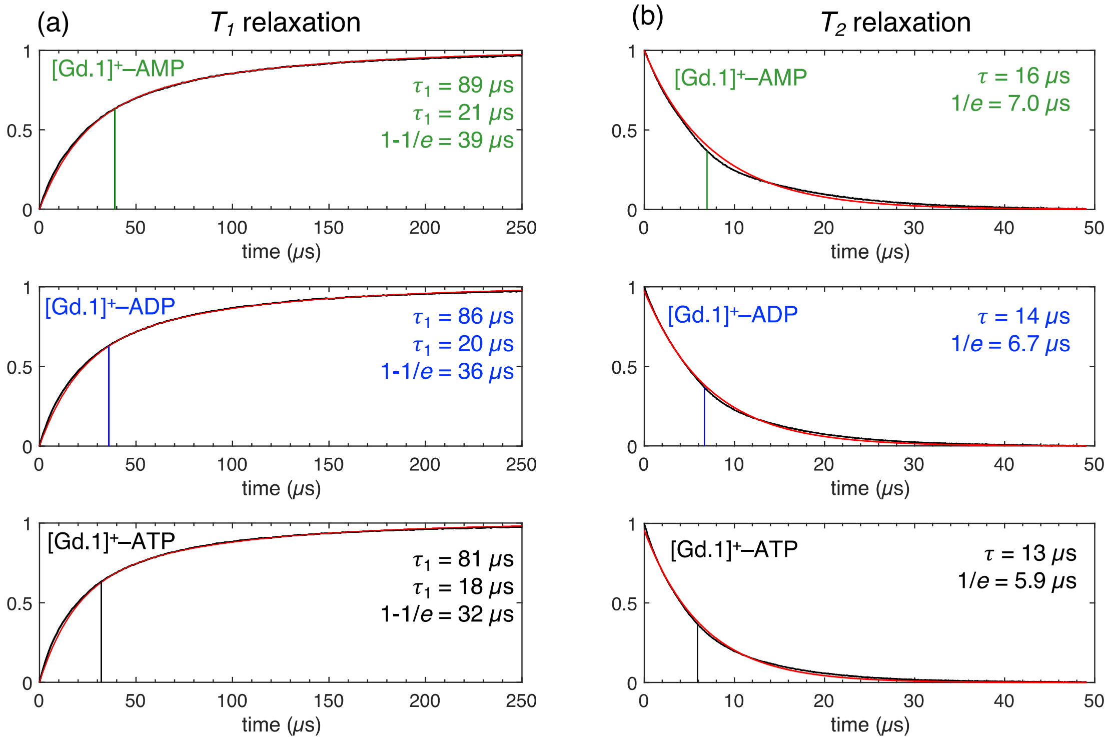

Figure S12. EPR relaxation data (black traces) and fits (red traces), according to the fitting parameters listed in Table S5. The coloured solid lines indicate the $\mathbf{1-1/e}$ and $\mathbf{1/e}$ inversion recovery *(T_1)* and decay (*T_2*) rates respectively. The data is for the **[Gd.1]⁺** complex bound to the different anions AMP, ADP, and ATP in 1:10 Gd(III) complex to anion concentration ratios. Panel a) shows the T1 data, while panel b) displays the T2 data.

EPR Line Broadening.

In the main text we showed how the EPR lineshape changes as a result of binding with different nucleoside phosphates. A reasonable fit to the structure of these line shapes could not be reasonably achieved, and we suggest that the lineshape may be explained by a superposition of signals from differently bound states. Fig. S13 shows an attempt to fit to the unbound [**Gd.1**]$^+$ complex. As with the anion-bound complexes, a perfect fit to the unbound complex data could not be obtained, although we found that the lineshape of the central $-1/2 \leftrightarrow 1/2$ could be reasonably fit by using a small rhombic D-tensor (zero field splitting) and a large distribution in the ZFS parameters (large D-Strain)). In the simulation in Fig. S13 a large D-strain on both the D and E values of 400 and 40 MHz respectively was used. In practice however, the fitting attempts only indicated that the ZFS distribution (i.e. strain) must be large in order to fit the central transition lineshape and capture the broadness of the underlying higher-spin transition envelope, but an exact value cannot be reliably determined.

We also found that the lineshape was dependent on the solvation conditions – the [**Gd.1**]+ dissolves better in higher concentrations of deuterated methanol (MeOD) and lower concentrations of D_2O, which indeed manifests in narrowing of the EPR lineshape. However high concentrations of MeOD lead to difficulties freezing the sample at sub liquid N_2 levels in narrow W-band capillaries (the tubes would shatter upon freezing), therefore final molar concentrations of MeOD in the EPR samples was 25%.

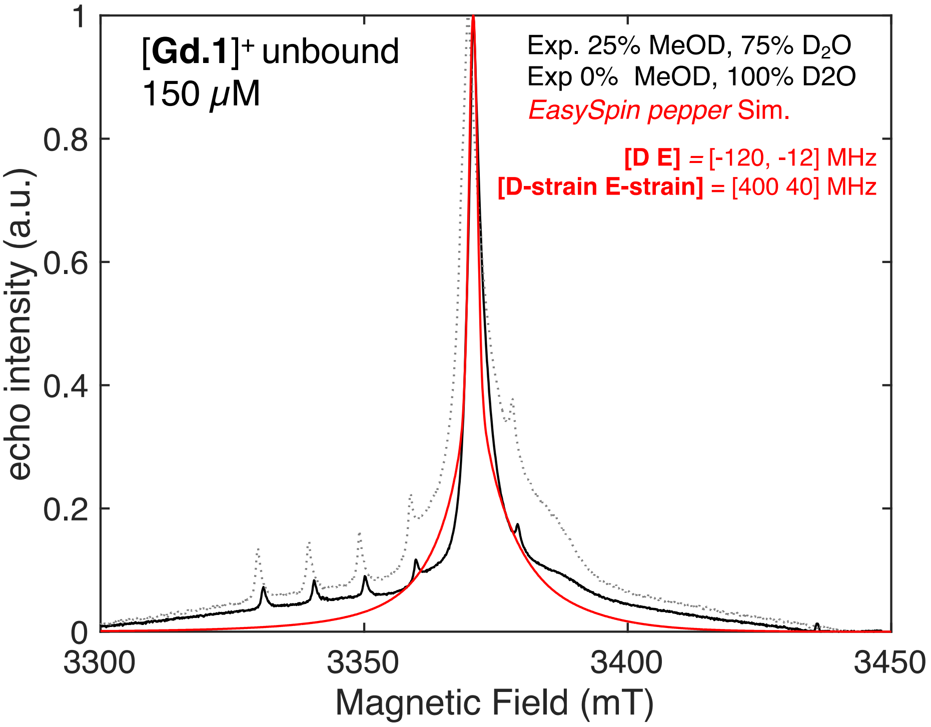

Figure S13. Experimental echo-detected lineshape spectra of the unbound [**Gd.1**]$^+$ complex in 25% MeOD solvent (black trace) and 100% D_2O solvent (grey dotted trace). The best fit simulation performed using the *EasySpin*[3] solid state simulation routine *pepper* is shown in red and annotated with the best-fit ZFS parameters. The sharp small features overlaying the Gd linewidth are background Mn(II) hyperfine signals.

^{31}P ENDOR Measured off the Central Line

To confirm that the line broadening of the [**Gd.1**]$^{+}$–ADP and ATP spectra was not due to an increased population of the transitions involving higher spin states (e.g. $\frac{1}{2}\leftrightarrow\frac{3}{2}$ or $-\frac{3}{2}\leftrightarrow-\frac{1}{2}$ transitions), we performed microwave pulse nutation measurements and ^{31}P Mims ENDOR measurements on the high field edge of the EPR line, to excite the broadened shoulder region of the [Gd.1]$^{+}$–ATP spectrum. A π/2 pulse of 20 ns has an excitation bandwidth of 50 MHz, so this is well separated from maximum of the Gd(III) central line. Figure S14a shows the proportion of spins used for detection, while Fig.S14b and S15 show the mw nutation and ENDOR spectra respectively. A contribution from higher spin transitions should increase the microwave nutation frequency, however at both the central and +7 mT positions, the mw nutation appears the same.

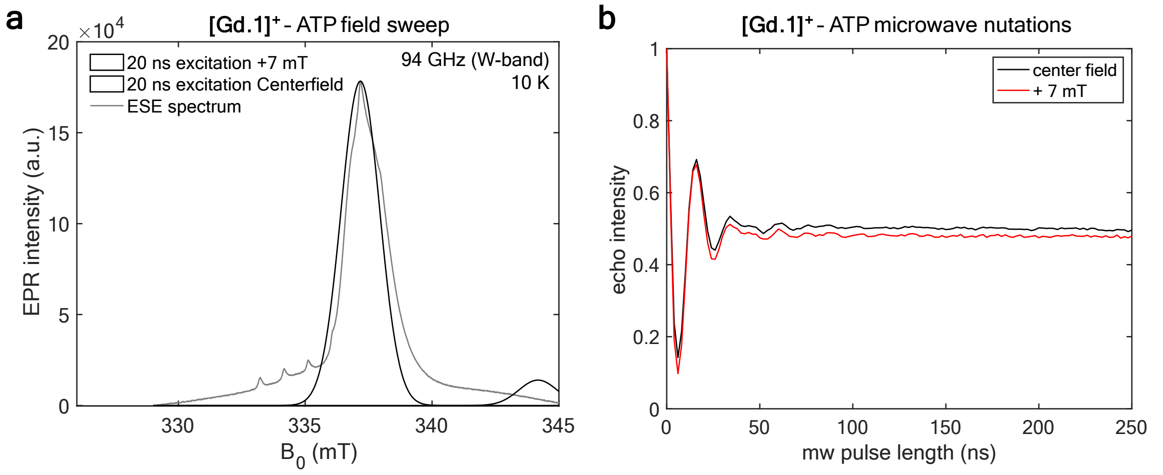

Figure S14. Off-centre field measurements of the [**Gd.1**]$^{+}$–ATP complex. (a) Electron-spin echo field sweep measurement of the [**Gd.1**]$^{+}$–ATP EPR line overlayed with the theoretical excitation profile of a 20 ns π/2 pulse at the centre field (red shading) or 7 mT above the centre field (grey shading). (b) Corresponding microwave nutations at the centre field (red trace) and 7 mT above the centre field (black trace), to test for contributions from higher spin transitions which should resulting a faster nutation frequency.

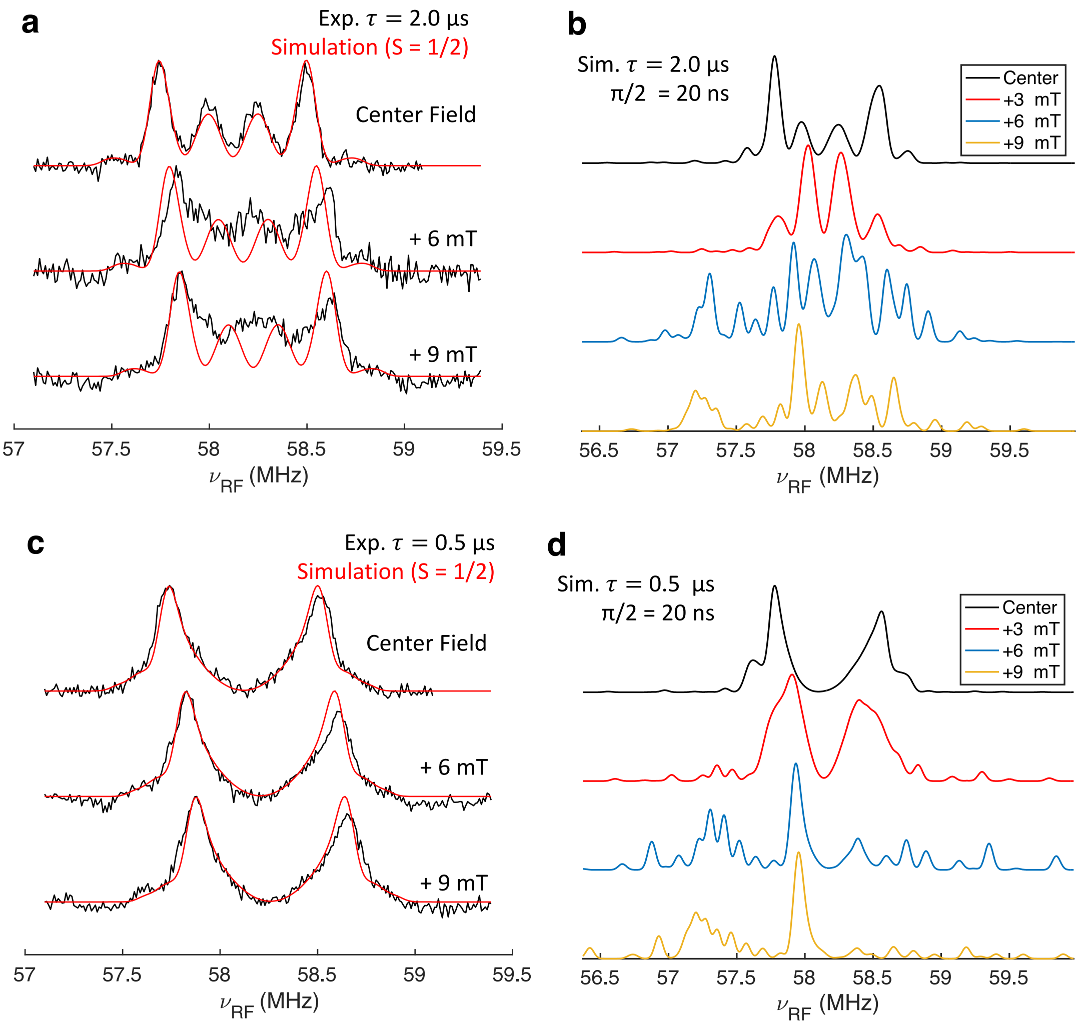

Figure S15. Simulation of experimental ^{31}P ENDOR spectra (black traces) measured by exciting either the centre of the EPR line, or at +6 mT or +9 mT field offsets off the central line. Panels a and c show the experimental $\boldsymbol{\tau} =$ 2.0 μs and $\boldsymbol{\tau} =$ 0.5 μs data overlayed with the simulation for an S = 1/2, I =^{31}P system (i.e. considering only the $\mathbf{-1/2 \leftrightarrow 1/2}$ EPR transition). Panels c and d show *EasySpin* Mims *saffron* simulations comparing the theoretical lineshape at different field offsets for a 20 ns exciting opulse with an excitation bandwidth of 50 MHz on a theoretical ZFS-broadened Gd^{3+} EPR spectrum (S = 7/2, D = 1000 MHz, E = 200 MHz).

In the ENDOR spectra, a large contribution of the higher spin transitions would result in additional, larger splitting and decreased relative intensity of the central ENDOR feature. This is seen in Figures S15 b and d, which show Mims *saffron EasySpin*[3] simulations incorporating an excitation bandwidth for a 20 ns pulse centred at the indicated field offsets on a theoretical Gd^{3+} (S = 7/2) spectrum with a large ZFS of D =1000 MHz, E = 200 MHz. At higher field offsets a larger contribution of the $1/2 \leftrightarrow 3/2$, $3/2 \leftrightarrow 5/2$ and $5/2 \leftrightarrow 7/2$ transitions are captured. This change in lineshape is not the case for our data, where the overall ENDOR line is not strongly dependent on the excitation field position, suggesting the broad lineshape we see is mainly due to the broadening of the $-1/2 \leftrightarrow 1/2$ line. However, we do observe that off-centre excitation does result in some change in the blindspotting pattern, mostly clearly seen for the $\tau =$ 2.0 μs value. Exciting the edge of the EPR line at higher field offsets most likely results in selecting in only a small subset of orientations, and therefore does not capture the complete ENDOR powder pattern. We note that for a broad $-1/2 \leftrightarrow 1/2$ transition

incomplete excitation of the Pake pattern also likely occurs when measuring at the centre field (Figure S14a) but to a lesser extent.

Complete τ-dependent ^{31}P ENDOR Fits

In the main text we show the summed spectra for the individual ^{31}P ENDOR spectra measured for each sample at different interpulse delay times τ to overcome blindspotting artefacts and capture the full ENDOR Pake pattern. In Fig. S16-S18 we show the complete τ-dependence of the measured data along with the simulation fits. ^{31}P Mims ENDOR simulations were performed using the *EasySpin* package *saffron*,[3] and the τ-varied spectra were fit simultaneously (minimising the total standard deviation of all fits to data sets), with the same spin system parameters across the data set. Either one or two Gd(III)-^{31}P coupling tensors were fit to the data. The complete list of parameters determined for the best-fit simulations is detailed in Table 4 of the main text.

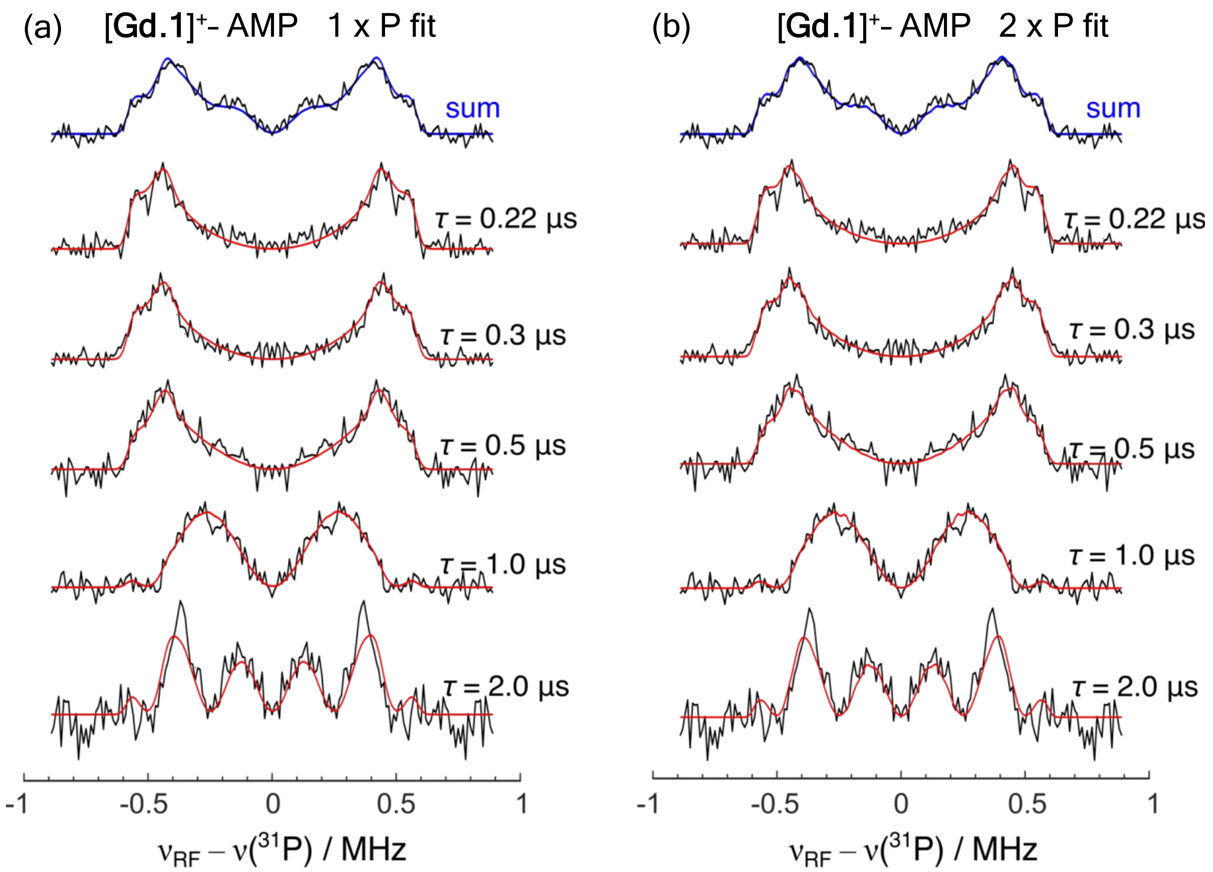

Figure S16. ENDOR data (black traces) and simulations (red traces) of AMP bound to **[Gd.1]+** in a 10:1 molar ratio. The spectra are measured at different interpulse delay times $\boldsymbol{\tau}$ and summed (blue trace). The fitting procedure minimises the fit to all $\boldsymbol{\tau}$ data simultaneously. Panel a) assumed one phosphorus couplings in the simulation fit, while b) assumes two ^{31}P couplings.

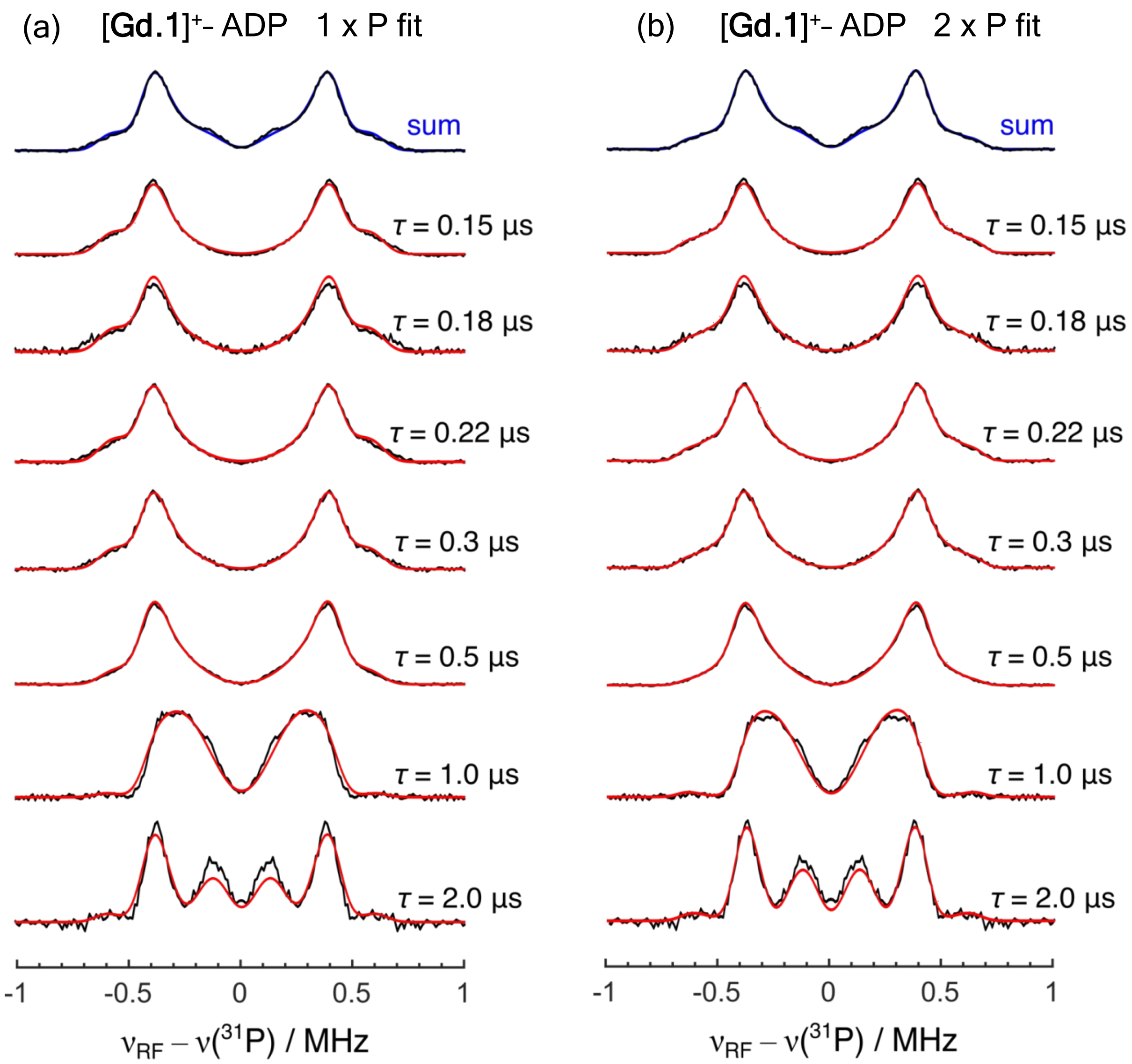

Figure S17. ENDOR data (black traces) and simulations (red traces) of ADP bound to **[Gd.1]⁺** in a 10:1 molar ratio. The spectra are measured at different interpulse delay times τ and summed (blue trace). The fitting procedure minimises the fit to all τ data simulataneously. Panel a) assumed one phosphorus couplings in the simulation fit, while b) assumes two ^{31}P couplings.

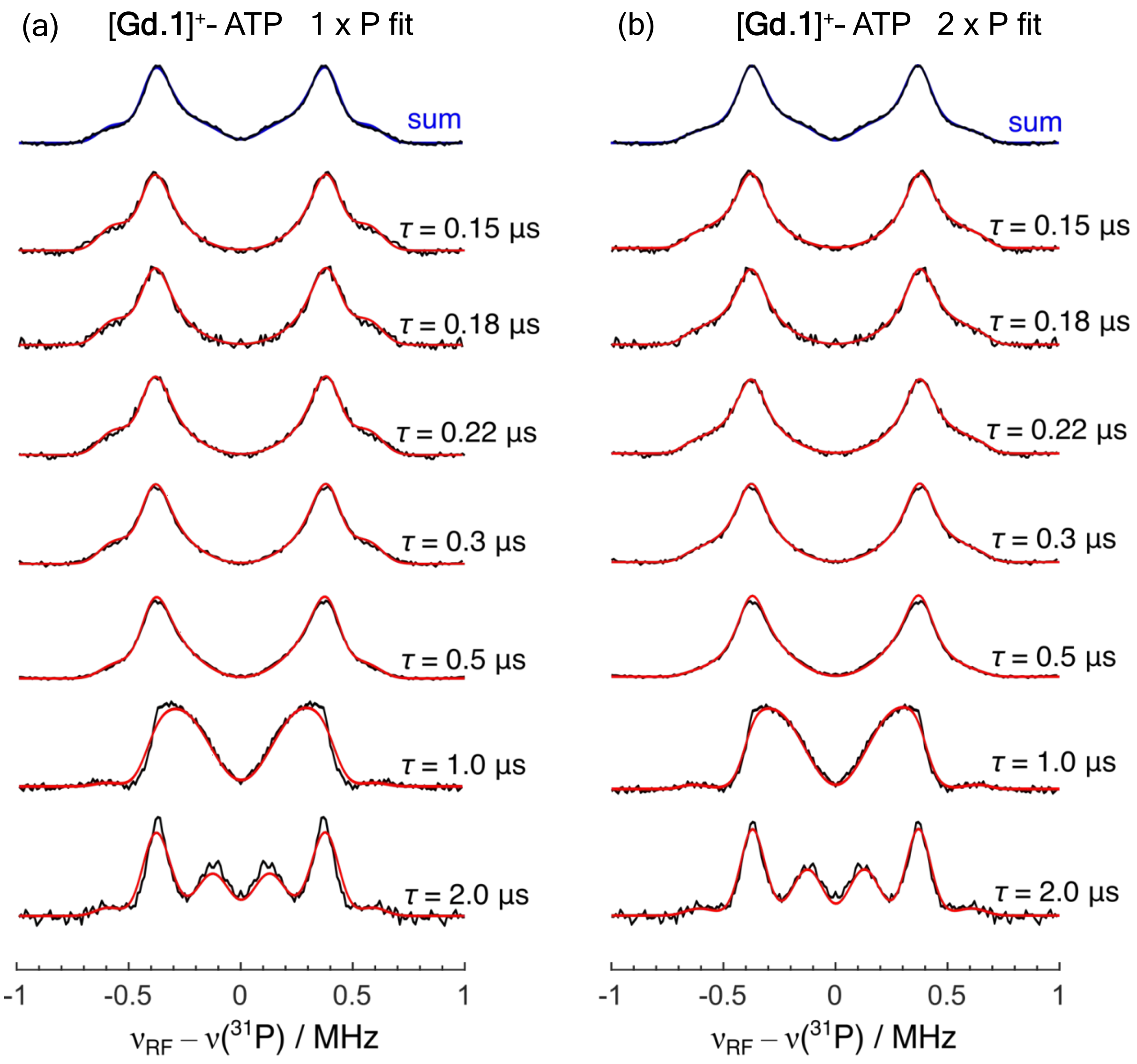

Figure S18. ENDOR data (black traces) and simulations (red traces) of ATP bound to **[Gd.1]**$^+$ in a 10:1 molar ratio. The spectra are measured at different interpulse delay times τ and summed (blue trace). The fitting procedure minimises the fit to all τ data simultaneously. Panel a) assumed one phosphorus couplings in the simulation fit, while b) assumes two ^{31}P couplings.

References

1. Hewitt, S. H.; Macey, G.; Mailhot, R.; Elsegood, M. R. J.; Duarte, F.; Kenwright, A. M.; Butler, S. J., Tuning the anion binding properties of lanthanide receptors to discriminate nucleoside phosphates in a sensing array. *Chem. Sci.* **2020,** *11* (11), 3619-3628.

2. Butler, S. J., Quantitative determination of fluoride in pure water using luminescent europium complexes. *Chem. Commun.* **2015,** *51* (54), 10879-10882.

3. Stoll, S.; Schweiger, A., EasySpin, a comprehensive software package for spectral simulation and analysis in EPR. *J. Magn. Reson.* **2006,** *178* (1), 42-55.
